# Importance of N_2_-Fixation on the Productivity at the North-Western Azores Current/Front System, and the Abundance of Diazotrophic Unicellular Cyanobacteria

**DOI:** 10.1371/journal.pone.0150827

**Published:** 2016-03-09

**Authors:** Virginie Riou, Debany Fonseca-Batista, Arnout Roukaerts, Isabelle C. Biegala, Shree Ram Prakya, Clara Magalhães Loureiro, Mariana Santos, Angel E. Muniz-Piniella, Mara Schmiing, Marc Elskens, Natacha Brion, M. Ana Martins, Frank Dehairs

**Affiliations:** 1 Aix-Marseille Université, Mediterranean Institute of Oceanography (MIO), UM 110 CNRS/INSU, IRD, 13288 Marseille, Université du Sud Toulon-Var, 83957, La Garde, France; 2 IMAR—Institute of Marine Research, Centre of IMAR at the University of the Azores, Horta, Portugal; 3 Analytical, Environmental and Geo-Chemistry & Earth System Sciences, Vrije Universiteit Brussel, Brussels, Belgium; 4 CIBIO, Research Center in Biodiversity and Genetic Resources, InBIO Associated Laboratory, Department of Oceanography and Fisheries, Horta, Portugal; 5 IPMA, I.P.—Portuguese Institute of Ocean and Atmosphere, Lisbon, Portugal; 6 MARE—Marine and Environmental Sciences Centre, Lisbon, Portugal; 7 DOP/UAz – Department of Oceanography and Fisheries, University of the Azores, Azores, Portugal; University of New South Wales, AUSTRALIA

## Abstract

To understand the impact of the northwestern Azores Current Front (NW-AzC/AzF) system on HCO_3_^−^-and N_2_-fixation activities and unicellular diazotrophic cyanobacteria (UCYN) distribution, we combined geochemical and biological approaches from the oligotrophic surface to upper mesopelagic waters. N_2_-fixation was observed to sustain 45–85% of the HCO_3_^−^-fixation in the picoplanktonic fraction performing 47% of the total C-fixation at the deep chlorophyll maximum north and south of the AzF. N_2_-fixation rates as high as 10.9 μmol N m^-3^ d^-1^ and surface nitrate δ^15^N as low as 2.7‰ were found in the warm (18–24°C), most saline (36.5–37.0) and least productive waters south of the AzF, where UCYN were the least abundant. However, picoplanktonic UCYN abundances up to 55 cells mL^-1^ were found at 45–200m depths in the coolest nutrient-rich waters north of the AzF. In this area, N_2_-fixation rates up to 4.5 μmol N m^-3^ d^-1^ were detected, associated with depth-integrated H^13^CO_3_^−^-fixation rates at least 50% higher than observed south of the AzF. The numerous eddies generated at the NW-AzC/AzF seem to enhance exchanges of plankton between water masses, as well as vertical and horizontal diapycnal diffusion of nutrients, whose increase probably enhances the growth of diazotrophs and the productivity of C-fixers.

## 1. Introduction

The increase in atmospheric CO_2_ concentration has stressed the need to quantify the transfer of CO_2_ by the marine biological carbon pump to the deep sea, where it can be trapped for centuries [1]. Although iron (Fe) and phosphorus (P) can limit C-fixation in some regions of the ocean, nitrogen (N) is limiting or close to limiting in most of the oligotrophic oceans [2]. Once all the nitrate and nitrite have been used by phytoplankton in the euphotic zone, new primary production at the surface is only possible if N_2_-fixation occurs or if new nitrate sources appear. While upwelling provides new nitrate for phytoplankton growth, it also delivers deep ocean CO_2_, leading to less atmospheric CO_2_ sequestration by the biological pump. On the other hand, if new N from N_2_-fixation is added to surface waters, net atmospheric CO_2_ sequestration into export production occurs [3].

In the N depleted North Atlantic (Sub)Tropical gyre (NAST), Fe-enhanced surface N_2_-fixation, which is limited by P availability, was estimated to add the equivalent of 50–180% of the deep ocean nitrate flux into the euphotic zone [4,5]. However, the geochemical, biological, and numerical modeling estimates vary widely [6] and there is an urgent need to evaluate the impact of N_2_-fixation on sea surface productivity with more accuracy to be able to predict the future efficiency of the biological carbon pump.

**Fig 1:**
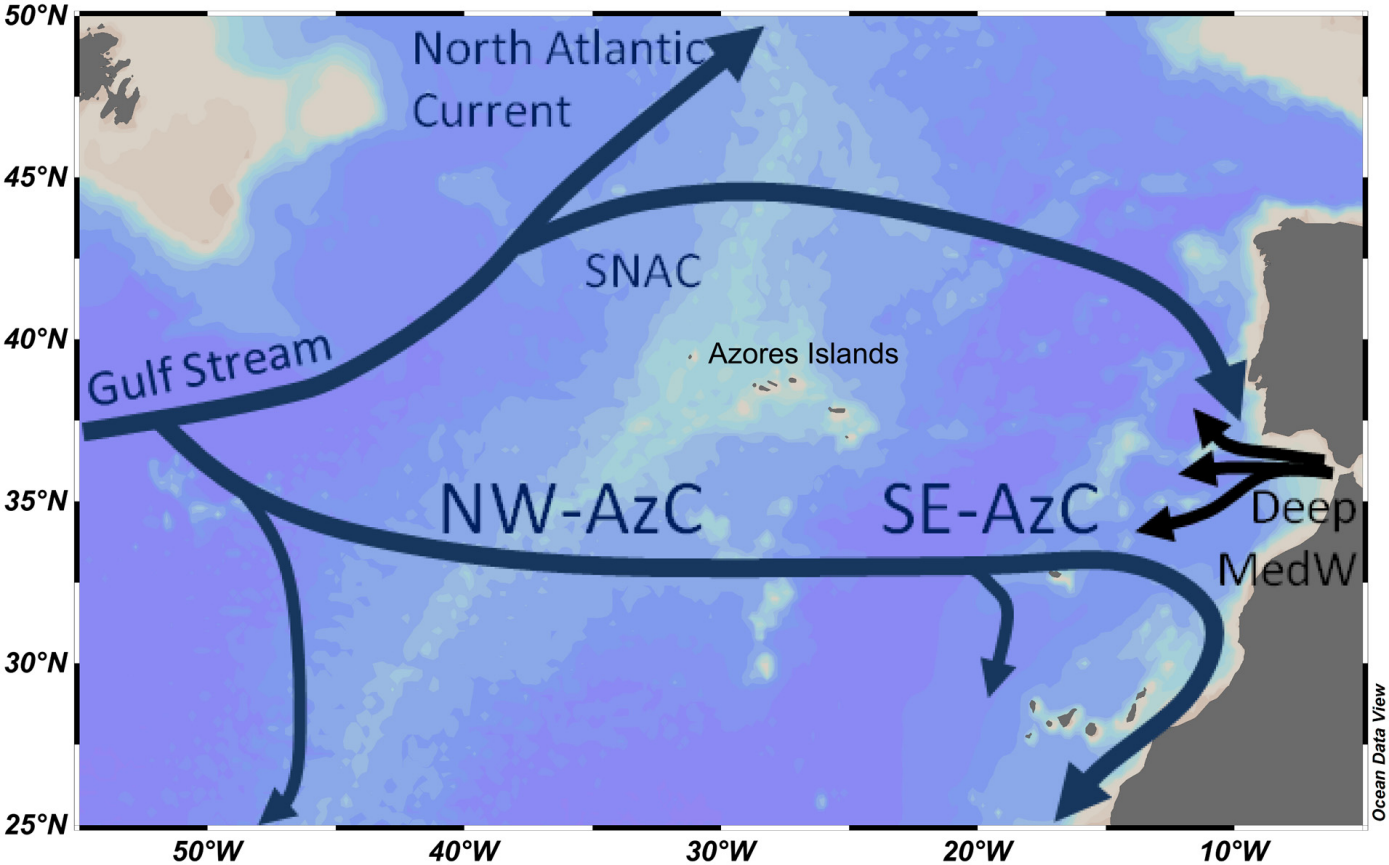
Map of the study area in the North Atlantic showing the main circulation patterns. The approximate locations of currents were re-drawn from [10,15]. SNAC: Southern branch of the North Atlantic Current. AzC: Azores Current. Deep MedW: Mediterranean outflow.

Hydrographic fronts are known to affect biological activity and primary productivity, and therefore potentially gas exchanges at the ocean–atmosphere interface. The Azores Front (AzF) extends over the whole width of the eastern NAST basin (Fig 1). It marks the northern border of the Azores Current (AzC, 32–36°N with a main axis ~34°N, [7], Fig 1), separating cold, Eastern North Atlantic Central Waters, from the more saline, warmer 18°C Mode Water (18MW), a homogenous, well mixed water body associated with the Gulf Stream extension [8]. Previous studies showed that the North West-AzF area (NW-AzF, 30–40°W) differs markedly from the south-eastern border of the AzC-AzF system (SE-AzF, 20–25°W) regarding water mass structure, with the 18MW detected only until 30°W [9]. Although N_2_-fixation was estimated to contribute 40% of the carbon export at the SE-AzF [10], its importance in the NW-AzF area is still unknown, and direct primary production measurements are missing for this area. The passage of the AzC across the Mid-Atlantic Ridge moreover increases the formation of eddies [11,12], which are known to affect both C- and N_2_-fixations [13,14]. It is therefore crucial to assess the relative importance and spatial variability of both processes *in situ* at the NW-AzC/AzF system.

Various methods are available to assess the amount of N_2_-fixation associated with sea surface productivity. Geochemical measurements generally reveal the weekly to seasonal integration of biological and physical processes. N_2_-fixation injects new N with low δ^15^N (-2 to 0‰, e.g. [16]) into the organic matter (OM), compared to upwelled deep ocean nitrate (δ^15^N = 5.0±0.5‰; [17]). Low δ^15^N signatures from particulate OM have been used to estimate the input of N_2_-fixation to microbial biomass in the NAST [18].

An increase of N_2_-fixation in the OM, followed by remineralisation and nitrification producing new nitrate, will result in a local decrease in δ^15^N_NO3_. Additional information can therefore be obtained from nitrate isotopic composition, which is unaffected by the OM stoichiometry. A decrease in δ^15^N_NO3_ may however be masked (i) by concurrent nitrate assimilation, which leads to nitrate ^15^N enrichment, and/or (ii) by mixing with abundant deep nitrate. Simultaneous analysis of the δ^18^O_NO3_ signature may distinguish these processes. Contrary to δ^15^N_NO3_, the δ^18^O_NO3_ signal is not affected by the remineralised OM composition during nitrification [19], while nitrate assimilation will enrich both δ^15^N_NO3_ and δ^18^O_NO3_ signatures equally [20]. The assimilation of both deep and new nitrate will therefore result in similar δ^18^O signatures, while the difference in their δ^15^N signals will remain. Two recent studies that analysed the difference in δ^15^N_NO3_ and δ^18^O_NO3_ have revealed significant contribution of N_2_-fixation to NAST surface waters [10,21].

The geochemical indicators of N_2_-fixation (OM δ^15^N and nitrate δ^15^N/δ^18^O) can be complemented by daily-resolved activity measurements, following the incorporation of ^15^N_2_-tracer in particulate OM (net N_2_-fixation). The ^15^N_2_ “bubble-addition method” has allowed measuring *in situ* N_2_-fixation rates for the last two decades [22]. Recent studies, however, showed that this method under-estimates N_2_-fixation rates by 62% to 570% in comparison to the “dissolution method”, due to slow equilibration of the ^15^N_2_ gas bubble with natural dissolved N_2_ during incubation [23,24,25]. The magnitude of this under-estimation was observed to depend on the composition of the diazotrophic community. Buoyant *Trichodesmium* filamentous cyanobacteria that stay in contact with gas bubbles would thus be much less sensitive to the amount of dissolved ^15^N_2_ gas than filamentous *Richelia* cyanobacteria growing inside diatoms, diazotrophic unicellular cyanobacteria (UCYN), or *γ-Proteobacteria* [23,24,25].

Deciphering the composition of the diazotrophic community is also of high relevance to estimate the extent of their contribution to organic C export. While diatoms-*Richelia* associations are known to contribute directly to C export [26], *Trichodesmium* also fix CO_2_ but are not known to sink beyond the euphotic zone. They would therefore rather fuel upper ocean microbial production [27] and fertilize surface waters with new N, allowing carbon export by other species. UCYN may add as much new N to the global ocean as *Trichodesmium* [28], which was shown to add more N to the euphotic zone than the estimated vertical flux of deep sea nitrate in the tropical North Atlantic [5]. However, UCYN-A, one of the three groups identified to date, is unable to perform C-fixation and although cultured representatives of UCYN-B and C are obligate photoautotrophs, there is no evidence for their export to depth [29]. Predation, aggregation and association to calcifying or silicifying unicellular algae may, however, promote net C export and UCYN-B may excrete significant amounts of N and C to be used by surrounding larger phytoplankton (reviewed in [29]).

N_2_-fixation was believed to be negligible at northern latitudes of the mid-NAST [4] until significant activity was detected at 34–42°N [30]. The subtropical mid-Atlantic north of 30°N has been much less investigated than the lower latitudes for the diversity and activity of diazotrophs [25,31,32]. Cyanobacteria-like *nifH* genes represented almost half of the sequences amplified from samples collected in the Azores. While filamentous cyanobacteria have not been detected north of 30°N in the mid-NAST, UCYN-A appear to dominate south of the Azores [31,33,34]. UCYN distribution is, however, barely known in the NW-AzC/AzF area. At the northern boundary of the NAST, UCYN-A abundances of up to 150 cells mL^-1^ have been detected using a UCYN-A-specific probe for whole-cell ribosomal RNA tyramide signal amplified-fluorescence *in situ* hybridization (TSA-FISH) [33,30]. This powerful technique has also been used with the Nitro821 probe targeting the three UCYN phylotypes, which allowed recognizing the worldwide importance of the picoplanktonic UCYN-A as free-living cells, or associated with inert or living particles (e.g. [33,30,35,36,37,38]).

Since evidence for the presence of UCYN is growing for higher latitudes of the North Atlantic Ocean up to 42°N [33,30], it is important to assess their distribution, as well as the activity of the diazotrophic community using the “dissolution method” in that region. We focused the present study on the NW-AzF region, corresponding to the Mid-Atlantic 30–34°N latitude belt where information on C- and N_2_-fixation and UCYN distribution is limited. The upper ocean was explored down to 200m for (i) C- and N_2_-fixation activities with the “dissolution method”, and (ii) Nitro821-positive UCYN abundance by TSA-FISH. These biological data were complemented with nitrate isotopic signatures used as a geochemical tracer of N_2_-fixation, and linked to the physical-chemical conditions. This information is essential to constrain N_2_-fixation in the North Atlantic and to understand the parameters influencing UCYN density and activity *in situ*.

## 2. Materials and Methods

**Fig 2:**
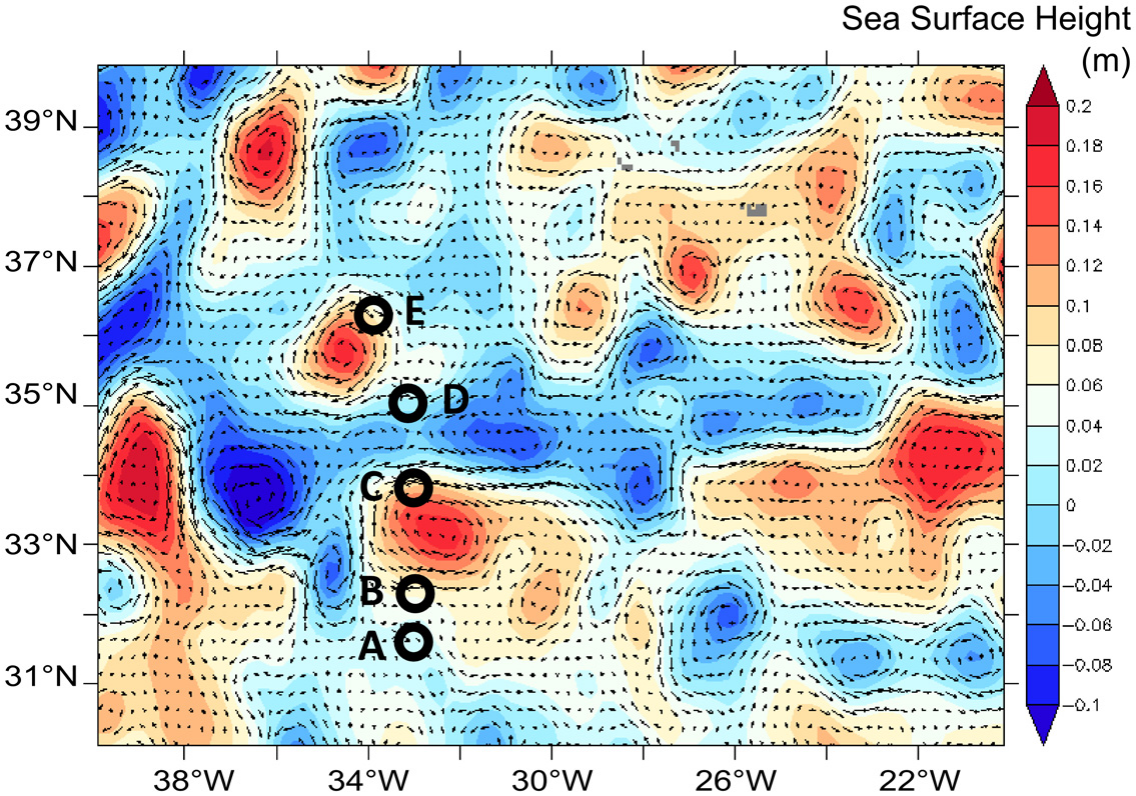
Sea Surface Height and geostrophic currents during the DIAPICNA cruise. Station locations (open circles) overlaid on AVISO altimetry-derived geostrophic currents centered between 26/07/2011 and 01/08/2011, with color scale indicating the Sea Surface Height (SSH) in m.

During the DIAPICNA cruise (25 July-3 August 2011) aboard the *NRP Dom Carlos I*, five stations (A-E) were sampled between 31.5°N-33.0°W and 36.2°N-33.9°W, with the authorization of the portuguese *Comissão Oceanografica Intersectorial–MCTES*. The station positions ensured sampling of both sides of the NW-AzC-AzF system close to the Mid-Atlantic Ridge, as identified from real-time AVISO satellite altimetry-derived mean geostrophic currents (Fig 2, S1B–S1F Fig).

### 2.1 Water-column physical and chemical characteristics

Temperature, salinity, chlorophyll (Chl) fluorescence, O_2_ saturation and turbidity profiles were obtained from a SBE-9plus CTD profiler coupled with WETlabs ECO-FLrtd-deep Chl fluorometer, SBE-43-dissolved O_2_ and SBE-911-turbidity sensors. Data processing and filtering were done using the Seasoft V2 software (Sea-Bird Electronics). The sensors were mounted on a SBE-32 Carousel together with 12 Niskin 2.5 L bottles used to sample seawater at 5, 25, 45, 75, 100, 125, 150, 175, 200, 250, 350 and 500m depths. For nutrient concentrations and nitrate isotopic signatures, quadruplicate 25 mL water samples were filtered onto sterile 0.45 μm porosity Acrodisc filters (Sterlitech), collected in 40 mL polypropylene vials (Nalgene) and immediately preserved at -20°C. Nitrate, ammonium and phosphate concentrations were determined back in the lab using a QuAAtro segmented flow automatic analyzing system (SEAL Analytical) with detection limits (d.l.) of 120, 120 and 140 nmol L^-1^, respectively. Concentrations of phosphate below the d.l. were determined on a 1 m Liquid Waveguide Capillary flow Cell (LWCC) with a QE65000 detector (Ocean Optics), down to 3 nmol L^-1^ concentrations (d.l.). N and O isotopic ratios of dissolved nitrate (>1 μmol L^-1^) were obtained with the “denitrifier method” [39,40] (see S1 Text).

### 2.2 Particle isotopic composition and C- and N_2_- fixation activities

Seawater samples were taken from each station at the surface (11–16m), above the Deep Chlorophyll Maximum (above DCM; 45–48m), at the DCM (86–112m) and in the upper mesopelagic zone (200–217m). A subsample of 4.5 L was immediately filtered for natural particulate organic carbon and nitrogen concentrations (POC/PN) and isotopic compositions (δ^13^C_POC_/δ^15^N_PN_). Rates of C- and N_2_-fixation were measured with the dissolved NaH^13^CO_3_ and ^15^N_2_ tracer method, as detailed in the supplementary material (S1 Text). A recent study showed that some batches of commercial ^15^N_2_ gas could contain ^15^N-labeled contaminants such as nitrate, nitrite and ammonium which could have greatly biased past estimates [41]. The influence of potential contaminants associated with the dissolution of the same ^15^N_2_ gas reference as used in the present study (Eurisotop ^15^N_2_ 98+ atom%), was tested in low nutrient waters amended with IAEA nitrate and ammonium reference compounds. No significant difference was detected in the δ^15^N signatures of the latter compounds, indicating that potential contamination of the gas used for the present study was negligible (Fonseca-Batista *et al*., unpublished data). Incubations were performed in duplicates in 4.5 L Nalgene polycarbonate bottles. These were filled to the very rim with the sample after enrichment with a NaH^13^CO_3_ (Eurisotop 99 atom%) spiking solution and 285 mL of degassed (by vacuum pumping under magnetic stirring) 0.2 μm-filtered low nutrient seawater (Osil) containing dissolved ^15^N_2_. Both tracers (Eurisotop 99 and +98 atom%, respectively) were added to reach theoretical final enrichments of 10 ^13^C atom% and 5 ^15^N atom%. The incubations were performed for 24 h in on-deck incubators flushed with flowing surface seawater at around 24°C, and wrapped in blue filters (Rosco), selected to simulate 0% (surface), 70% (subsurface), 97% (DCM) and 99.9% (200–217m depths) daylight attenuation, according to Piazena *et al*. [42]. At the end of the incubation period, aliquots were withdrawn from the samples under helium pressure to measure the Dissolved Inorganic Carbon (DIC) ^13^C atom% and dissolved N_2_^15^N atom%. The aliquots were transferred through the PTFE septa, to 12 mL exetainers (Labco) poisoned with HgCl_2_, and measured on a Flash EA 1112 Elemental Analyzer coupled to a DELTA V Isotope Ratio Mass Spectrometer via a Conflo III interface (EA-IRMS, Thermo Instruments) equipped with a custom made manual gas injection port (see S1 Text). Aliquots of the incubations were also filtered on 0.45 μm Acrodiscs and kept at -20°C for nutrient analysis. Natural and enriched particles were subsequently size-fractionated by serial filtration onto 25 mm diameter membranes of 3.0 μm and 0.3 μm porosities, made of silver (Sterlitech) and pre-combusted glass fiber (GF75, Advantec MFS Inc.), respectively. They were treated and analyzed for POC/PN and δ^13^C_POC_ and δ^15^N_PN_ using the EA-IRMS, as detailed in the S1 Text.

### 2.3 C- and N_2_- fixation rates and error calculations

C- and N_2_-uptakes (Uptake C and N in nmol L^-1^) were calculated as:

Uptake X =(Afinalparticle −At=0particle) × ConcentrationAfinalsubtrate −At=0particle (1)

where ^*final*^*A*_*particle*_ is the ^13^C or ^15^N atom% measured in the particles after incubation, ^*t = 0*^*A*_*particle*_ is the natural ^13^C or ^15^N atom% measured in particles without incubation, *Concentration* is the POC or PN content after incubation (μmol L^–1^) and ^*final*^*A*_*substrate*_ is the DIC ^13^C atom% or dissolved N_2_^15^N atom% after incubation. The assumption was made that the 24 h H^13^CO_3_^-^ and ^15^N_2_-fixation activities did not significantly affect A_DIC_ and A_N2_. Uptake rates (μmol X m^-3^ d^-1^) were obtained by dividing the uptakes with the incubation duration. Data correction and selection procedure is detailed in the S1 Text. All C-uptake rates were above background, while 70 of the 94 ^15^N_2_ uptake values were at or below background and were reported as d.l. (below detection).

### 2.4 Unicellular cyanobacterial diazotroph (UCYN) cellular abundance

Samples from the surface, above DCM, DCM and upper mesopelagic casts were size-fractionated on 47 mm diameter polycarbonate membranes (PCTE, Sterlitech) of different porosities connected in series. Filters of 0.2, 3 and 10 μm porosity were used to collect the cells present in 250 mL, 2 L and 4.5 L of the sample, respectively. Cell fixation, preservation and TSA-FISH were done according to [35] (detailed in S1 Text), hybridising16S rRNAs with the horseradish peroxidase (HRP) labeled Nitro821 probe (5’-CAAGCCACACCTAGTTTC-3’, ThermoFisherScientific GmbH) specific for UCYN [43]. The hybridized cells were stained with fluorescein-tyramide using the TSA system (TSA kit, PerkinElmer), followed by DNA counter-staining of all the cells using DAPI (4’,6’DiAmidino-2-PhenylIndole, Sigma-Aldrich). Nitro821 positive cells were counted with a 40X objective (NA 0.75N Plan Fluor WD 0.72mm, Nikon) on an epifluorescence *ECLIPSE 50i* microscope (Nikon) using a Halogen lamp (H65761, Orbitec) and dichroïc filters for DAPI (Excitation 365±10 nm, Emission 400 nm) and Fluorescein IsoThioCyanate (Ex. 480±40 nm, Em. 510 nm long pass). UCYN cells detected in the <3 μm and >3 μm size fractions were grouped into three categories: (1) free picoplanktonic cells (<3 μm), (2) picoplanktonic cells attached to particles or larger algae, and (3) nano-planktonic cells (3–10 μm). The entire surface of each 1/16^th^ filter portion was counted following [37], after we validated that it was representative for the whole sample. Validation was done by counting triplicate portions of the same sample, resulting in relatively low standard deviations (e.g. 24.45 ± 1.98 cell mL^-1^ in the <3 μm size fraction, see also [37]). Relatively high triplicate variability was observed for cell counts below 0.2 cell mL^-1^ (0.12 ± 0.09 cell mL^-1^ for the <3 μm size fraction; 0.09 ± 0.07 cell mL^-1^ for the >10 μm size fraction). Filters of all porosities (0.2, 3 and 10 μm) were counted, and the results for the 3 and 10 μm porosity filters were pooled to compare with the >3 μm fraction ^15^N_2_ uptake rates.

### 2.5 Statistical analysis and data availability

For each depth strata, pairplots were done for the response variables (POC, PN, C- and N_2_-fixation, Pico-UCYN abundance) and selected environmental variables (% surface photosynthetically active radiation-PAR, potential temperature, salinity, % O_2_ saturation, nitrate, phosphate, ammonium and silicate concentrations). The plots were done with the software R (version 3.1.3, [44]). In addition, the Spearman rank correlation coefficient was calculated for the same variables using a Bonferroni correction to account for multiple comparisons.

The metadata acquired during DIAPICNA are publically available from the Marine Data Archive at the Belgian VLIZ institute under the following DOI: http://dx.doi.org/10.14284/40

## 3. Results

### 3.1 Water-column physical properties across the NW Azores Current

**Fig 3:**
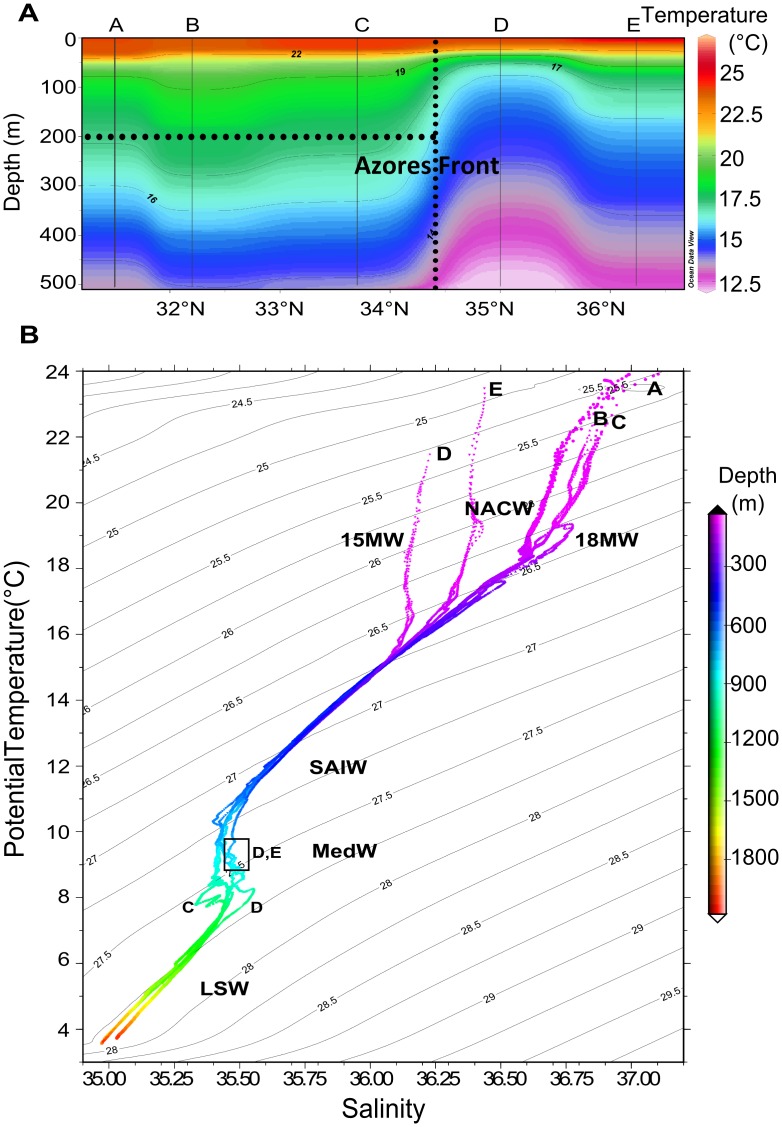
Physical parameters monitored along the DIAPICNA cruise track. (A) Temperature (°C) longitudinal cross-section, with the position of the Azores Front deduced from the 16°C isotherm at 200m depth (dotted lines); (B) TS diagrams for stations A-E, with overlaid σ_θ_ density isolines. NACW: North Atlantic Central Water; 15MW: 15°C Mode Water; 18MW: 18°C Mode Water; SAIW: SubArctic Intermediate Water; MedW: Mediterranean Water; LSW: Labrador Sea Water. The black box indicates possible increased influence of Mediterranean Waters at 800–1000m in stations D and E.

The AzF was located between stations C and D, as derived from the 16°C isotherm at 200m depth [11] (Fig 3A). Below 80m depth, the AzF separated warmer and more saline waters to the south, from cooler and fresher waters to the north. Isotherms and PAR (S2 Fig) reached deeper inside the AzC (stations B-C). Anticyclonic features drove downwelling at stations B and C, but also at station E (Fig 2; S1C, S1D and S1F Fig). On the contrary stations A south of the AzC and D north of the AzF, did not seem to be associated to any particular hydrological feature and may be considered as “reference” stations for the physical-chemical conditions met south and north of the NW-AzC, respectively.

Surface waters from 0–300m consisted of North Atlantic Central Water (NACW). South of the AzF (stations A-C), the surface T-S diagram patterns were typical of the 18°C Mode Water of subtropical origin (18MW, σ_ϴ_ = 26.5, Fig 3B). These waters were consistently more saline by 0.38–0.61 units than the northern NACW surface waters (stations D-E, S3A Fig). The T-S relationship at station D (0–100m) was characteristic of the 15°C Mode Water of subpolar origin (15MW, σ_ϴ_ = 26.9). Surface waters at station E were more saline (0–200m) and more than 1°C warmer down to 10m depth (25.2°C) than at all other stations (23.5–24.2°C). The euphotic zone, with its lower boundary at 1% of surface PAR, extended to 85–103m at station A, 106–112m at stations B and C, and 76–98m at stations D and E. The upper mixed layer reached down to 25–40m at station A, and shoaled towards the north (20–25m at stations B-C, and 5–13m at stations D-E). The seasonal thermocline also shoaled from 60m in the south to 30m northward. *In situ* Chl fluorescence profiles indicated the presence of DCM at 87–109m south of the AzF, and at 65–85m in stations D and E (S3B Fig). These DCM were associated with O_2_ concentrations close to saturation (S3C Fig).

Below 300m, two main layers were identified at all stations: (i) the main thermocline layer of the NACW (26.8<σ_ϴ_<27.2, 300–600m) and (ii) intermediate levels (27.2<σ_ϴ_<27.9) of the SubArctic Intermediate Water (500–800m, SAIW), Mediterranean Water (MedW) and Labrador Sea Water. The T-S diagram from station E appeared to be more influenced by MedW at about 800–1000m (as in station D, Fig 3B), although there is no conclusive evidence that the nearby anticyclonic eddy was in fact a MedW eddy (no O_2_ minimum layer expected from MedW). In addition, due to the persistence of this eddy in the region, it is expected that waters from station E have received some influence from waters further south (AzC and 18MW, S1A Fig).

### 3.2 Nutrient concentrations

**Fig 4:**
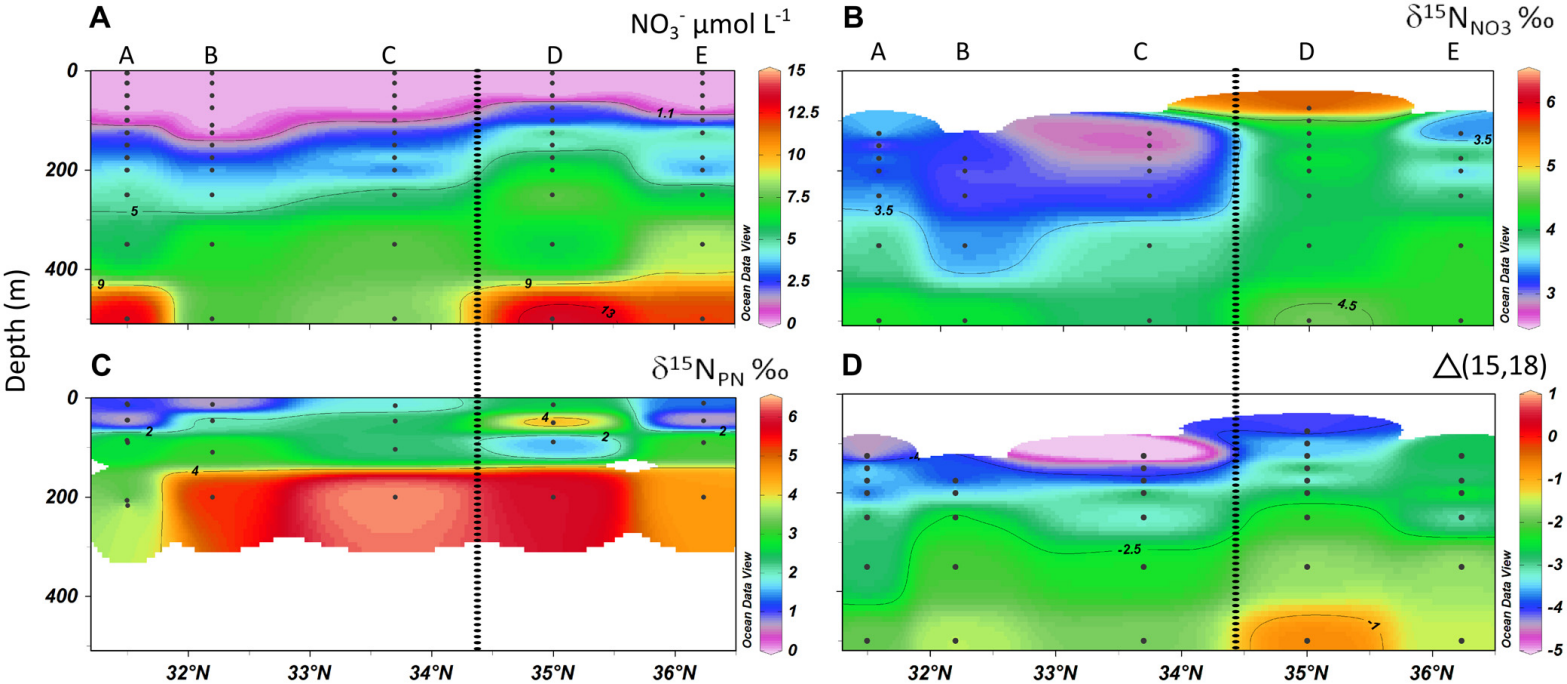
Geochemical tracers of N_2_-fixation along the DIAPICNA cruise transect. Longitudinal cross-sections of (A) nitrate concentration, (B) δ^15^N_NO3_ signal, (C) PN isotopic signature and (D) Δ(15,18)—see eq (2). The position of the Azores Front is indicated by a dashed line, and station i.d. is given on top.

The top of the nutricline corresponded to the depth of the DCM (Fig 4A, S3B and S3D Fig). Above it, low phosphate (<30 nmol L^-1^) and nitrate (<d.l. = 120 nmol L^-1^) concentrations down to 75m (except at station D) indicated that the area was oligotrophic. Values measured 125–200m deep, respectively south (stations A-C) and north of the front (stations D-E), were 1.5–4.3 and 3.1–6.5 μmol L^-1^ for nitrate (except for station B at 125m: <d.l.) and 0.02–0.24 and 0.17–0.28 μmol L^-1^ for phosphate.

The nutricline deepened at station B (close to 32°N), with phosphate levels below the d.l. down to 200m depth, associated with lower nitrate concentrations. In contrast, the uplift of isotherms and isohalines at station D indicated the presence of deeper waters, richer in nutrients north of the AzF (Fig 3A, S3A Fig). Overall ammonium concentrations were below 0.27 μmol L^-1^, showing no real trend with depth (S3E Fig). Higher concentrations of up to 0.49 μmol L^-1^ were detected at station C (above and below the DCM), station D (75–125m) and station E (125–175m).

### 3.3 Particulate Nitrogen (PN) and nitrate natural isotopic compositions

In the euphotic zone (from the surface to the DCM), average natural δ^15^N_PN_ signatures of particles within the <3 μm and >3 μm size classes were 1.9±1.6‰ and 2.1±1.2‰, respectively (weighted means, Fig 4C). In <3 μm particles, they varied between -0.3 and +3.1‰ in stations A, B and E, were around +2.5‰ in station C (with an unrealistic high value of +11.0‰ at 47m, omitted from the weighted means calculations, and probably due to the presence of a large aggregate or copepod on the filter) and varied from +0.7 to +5.4‰ in station D. Particles >3 μm had δ^15^N_PN_ signatures ranging from +0.2 to +4.3‰ in all stations except station D, where they were rather constant at +2.2 to +3.2‰. In the upper mesopelagic (200–217m), suspended particles were relatively enriched in ^15^N (+4.3 to +14.0‰), except in particles >3 μm at stations A, B and D (-0.3 to +2.8‰, S1 Table).

**Fig 5:**
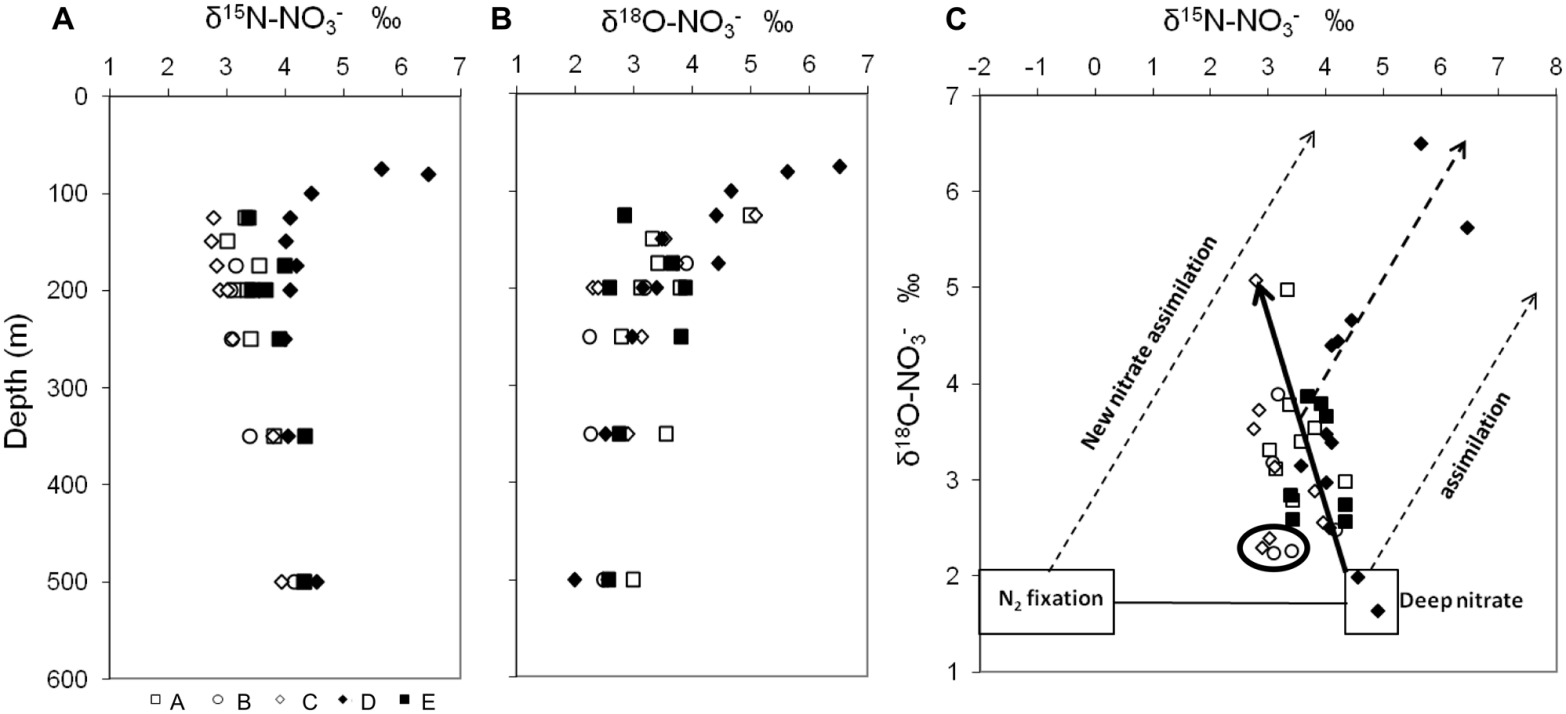
Nitrate N and O isotopic signatures. Nitrate δ^15^N (A), δ^18^O (B) depth profiles and relationship between δ^18^O and δ^15^N (C): open symbols mark southern stations (A: squares, B: circles, C: diamonds), filled symbols indicate northern stations (D: diamonds, E: squares). Circled data: exceptionally low δ^18^O and δ^15^N, respectively indicating low nitrate assimilation and the influence of ‘new’ nitrate derived from N_2_-fixation, at stations B (250m and 350m) and C (200m).

In the top 500m of the water column, nitrate δ^15^N signature was slightly depleted in ^15^N south of the AzF, in comparison to waters north of the front (Figs 4B and 5A). At 500m, δ^15^N_NO3_ signatures were close to 4.0‰ in all stations and decreased by around 1.0‰ from 500m to 200m depth (δ^15^N_NO3_ = 2.9–3.6‰). The only exception was in station D, where δ^15^N_NO3_ decreased by <0.5‰ towards 200m (δ^15^N_NO3_ = 3.6–4.0‰) and increased to 5.6–6.4‰ in the DCM. At these shallower depths, values were generally lower south (2.7–3.3‰) than north of the AzF (3.4–4.0‰ below the DCM at station E), although larger fluctuations were observed among stations. In contrast, values of δ^18^O_NO3_ displayed a similar trend south and north of the AzF (Fig 5B). They ranged from 2.0–3.9‰ between 500m and 200m, increased to 3.4–5.1‰ above 200m (except at station E DCM: 2.8‰), and even reached 6.5‰ at station D (75m). The δ^18^O_NO3_ was strongly negatively correlated with nitrate concentrations over the 75–1900m depth range at station D only (δ^18^O_NO3_ = 6.4 e^-0.1[NO3-]^, r² = 0.82). At station E above the Rainbow hydrothermal vents, deep-water (1900m) nitrate isotopic signatures (δ^15^N_NO3_ = 6.2‰, δ^18^O_NO3_ = 0.5‰) differed from those at station D (δ^15^N_NO3_ = 4.9‰, δ^18^O_NO3_ = 1.6‰).

The nitrate N-to-O isotope anomaly, relative to a 1:1 relationship expected from pure nitrate assimilation following a fractionation relationship with a (^15^ε/^18^ε) slope of 1, was calculated using the following equation [19]:

Δ(15,18)= (δ15N−δ15Nm)− ε15ε18×(δ18O−δ18Om). (2)

where δ^15^N_m_ and δ^18^O_m_ were assigned values of 4.9‰ and 1.6‰, respectively, measured in station D 1900m deep water. A negative Δ(15, 18) was observed in the upper 500m along the whole transect, indicating a decrease in nitrate δ^15^N relative to the δ^18^O at stations A, B, C and E, and a minor increase of nitrate δ^15^N relative to the δ^18^O from 150m depth upwards in station D (Figs 4D and 5C).

### 3.4 C- and N_2_-fixation rates

**Fig 6:**
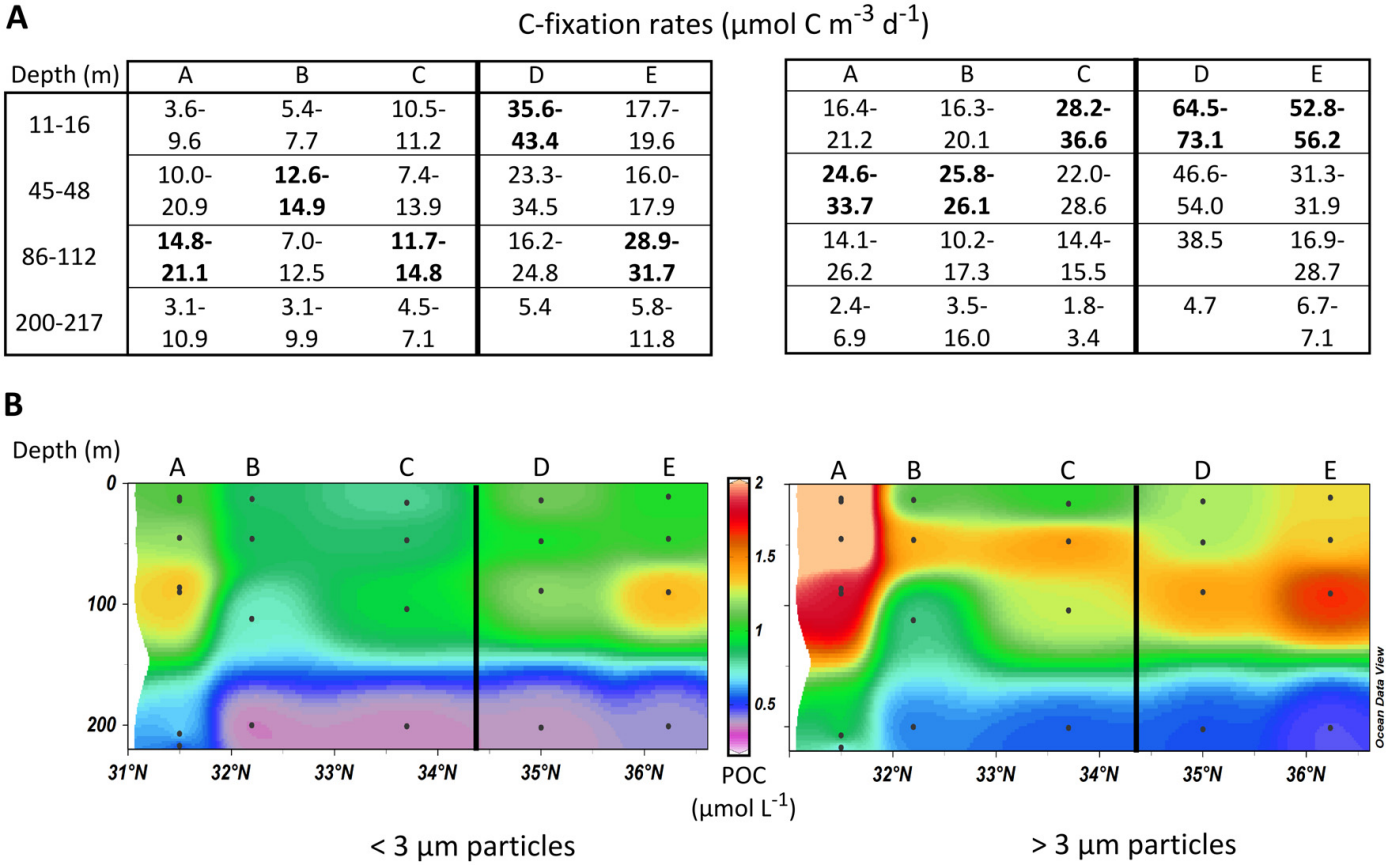
Distribution of Carbon fixation and Organic Carbon in <3 μm and >3 μm large particles. (A) C-fixation rates (μmol m^-3^ d^-1^), and (B) longitudinal cross-sections of POC (μmol L^-1^) depending on the particle size fraction (left: <3 μm; right: >3 μm). The Azores Front position is marked by a bold line.

The C-fixation rates in <3 μm and >3 μm size fractions are presented in Fig 6A. Measured ^13^C enrichment of DIC at the end of the incubations ranged from 8.1 to 11.2 atom% ^13^C (S2 Table). At the southernmost stations, the highest total rates (>3μm + <3μm) were observed above the DCM (stations A-B), whereas they were detected closer to the surface north of the AzC (11–16m, stations C-E). The picoplanktonic fraction (<3μm) accounted on average for 37% of the total production. It was most important in deeper layers, representing 47% of total production in the DCM and 55% in the upper mesopelagic. Station B, located inside the AzC, had the least productive waters. Waters were markedly more productive north (Stations E-D: 106–123 mg C m^-^² d^-1^) than south of the front (Stations A-C: 60–77 mg C m^-^² d^-1^) when C-fixation rates were integrated over the first 200m of water column. However, station A presented the highest POC concentrations in both size fractions, with values at 200m exceeding those at station D by a factor of 2 (Fig 6B).

**Fig 7:**
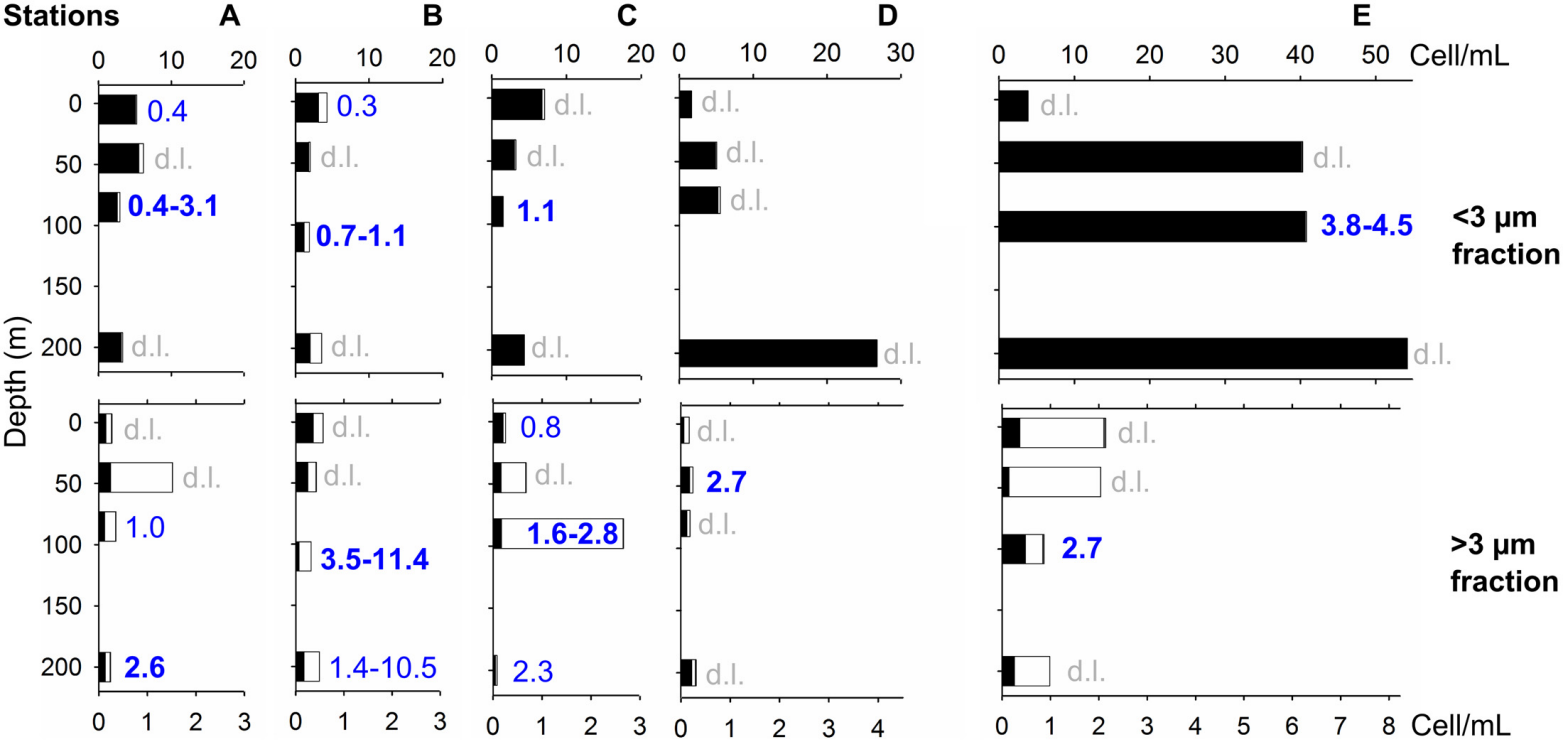
N_2_-fixation rates and UCYN abundance in <3 μm and >3 μm particles. Top panels: <3 μm particles; bottom panels: >3μm particles. For each station and depth, N_2_-fixation rates (μmol m^-3^ d^-1^) measured in each size fraction are indicated in blue (d.l. indicate measurements below the detection limit). Black bars indicate free pico-UCYN, white bars for associated pico-UCYN and grey bars for large pico- or nano-UCYN (in cell mL^-1^).

The highest N_2_-fixation rates for both size fractions were generally observed in waters collected at the DCM, except at station D (Fig 7). South of the AzF (stations A-C), high rates were measured in upper mesopelagic water >3 μm particles, with values increasing slightly in water samples taken at night in Station A (S3 Table). Preliminary tests of ^15^N_2_ enrichment prior to the cruise indicated that a final incubation enrichment level of 5.0 ^15^N atom% could be achieved and remain stable over a period of 9 days (S1 Text). However, this enrichment decreased to 0.4–1.0 ^15^N atom% for samples incubated during the cruise (see S1 Text and S1 Table, S3 Table). The Teflon-lined silicon septa used to seal the serum bottles with ^15^N_2_ spike was observed to not remain gas tight over prolonged periods. This caused loss of ^15^N_2_ during transportation and storage before the cruise. This problem was solved *a posteriori*, using bromobuthyl septa that avoided the loss of ^15^N_2_ enrichment for up to 2 months at 5–30°C. Significant uptake rates were obtained for one third of the incubation samples, ranging from 0.2±0.1 to 10.9±1.1 μmol N m^-3^ d^-1^, despite the low ^15^N_2_ enrichment, data correction and a drastic selection procedure.

### 3.5 UCYN abundance

The 16S rRNA sequence clade targeted by Nitro821 contains subgroups making most of the currently known marine planktonic cyanobacteria that have retained the nitrogenase genes cluster [43,45]. Members of all three UCYN groups identified to date (A, B and C) as well as unknown members are labelled by this probe without distinction. Labelled cells were present at all stations, mostly in the <3 μm size fraction (94.0%, Fig 7). The UCYN detected on both 3 and 10 μm porosity filters therefore represented a minor contribution, and the concentrations obtained on both filters were pooled in Fig 7, to allow comparison with the 3 μm filters used for C- and N_2_-fixation rates measurements. Overall abundances ranged from 2–55 cells mL^-1^, with the highest counts recorded north of the AzF, at 45–200m depths. The UCYN community was largely dominated by free-living 0.7–2.0 μm sized pico-cyanobacteria (pico-UCYN, 96.9% of the <3 μm fraction), with overall low contributions of 2–3 μm sized pico-UCYN and of free living nano-UCYN (0.2% of the whole UCYN community, mainly in station A <3μm at 200m, stations B and C <3μm at 45m and station E >3μm at 5m). Particle-associated and intracellular pico-UCYN represented 3.0% of the targets in the <3 μm size fraction, and 72.9% in the >3 μm size fraction.

The distribution of pico-UCYN was more homogeneous throughout the water column south of the AzF (1–7 cells mL^-1^) than north of it (1–54 cells mL^-1^). To the south, the lowest cell concentrations were found in the DCM, although increased counts of associated pico-UCYN (3 cells mL^-1^) were detected at station C in the DCM >3 μm fraction. North of the front (stations D-E), small pico-UCYN abundances increased by one order of magnitude with depth (from 1–3 to 27–54 cells ml^-1^), reaching maximum numbers at 200m. Although our sampling protocol did not allow the quantitative recovery of filamentous cyanobacteria, two trichomes were detected in the DCM at station E, where a significant N_2_-fixation rate was also observed (2.7±0.6 μmol N m^-3^ d^-1^).

### 3.6 Physical-chemical controls on UCYN concentrations, C- and N_2_-fixations

Pairplots performed on data from the same depth range, avoiding the bias introduced by large surface-deep variations, showed that UCYN abundances below the surface were not linked to any of the physical-chemical parameters examined (Table 1, S4 Fig, S4 Table). At the sea surface (11–16 m depths), <3 μm UCYN abundances were influenced by variations in salinity only. At these depths, C-fixation in the <3 μm and >3 μm size fractions were correlated, and they were not linked to the physical-chemical conditions. Below the surface, variations of C-fixation in the smaller size fraction were linked to oxygen and salinity fluctuations above the DCM (45–48m depths), where they were both correlated to silicate concentrations. In the DCM (86–112 m depths), POC and PN concentrations in the <3 μm size fraction was strongly correlated with C-fixation rates and *in situ* Chl fluorescence. Chl itself increased with decreasing salinity, that in turn was positively correlated to temperature. Decreasing temperatures were associated with significantly higher phosphate concentrations. In the upper mesopelagic zone (200–217m depth), where nitrate and silicate concentrations were linked to the water mass physical properties, none of the variables (UCYN concentrations, C- and N_2_-fixations) appeared to respond to variations of the selected physical-chemical parameters. South of the AzF, C- and N_2_-fixation rates tended to be linearly correlated (although not significantly) in >3 μm large mesopelagic particles.

**Table 1:** Significant correlations between C-, N_2_-fixations, UCYN abundance and physical-chemical conditions.

	C-Fixation Activity		Correlation	N_2_-Fixation Activity		Correlation	UCYN abundance		Correlation
**Surf.**	C-Fix s	C-Fix L	0.95***			None	Pico-UCYN s	Sal	1.00***
**Above DCM**	C-Fix s	Sal	(-)0.89*			None			None
		O2	0.89*			None			None
**DCM**	C-Fix s	PNs	0.94**			None			None
		POCs	0.92**			None			None
**Mesopelagic**			None			None			None
			None			None			None

Significance of the Spearman correlation at p<0.001, p<0.01 and p<0.05 are shown with ***, ** and *, respectively. O_2_: oxygen saturation %; Temp: potential temperature; Sal: salinity; PAR: % surface photoactive radiation; Chl: in situ Chlorophyll fluorescence. POC, PN: Particulate Organic Carbon, Nitrogen concentrations; C-Fix, N_2_-Fix: C- and N_2_-fixation and Pico-UCYN abundance are examined for the small (<3 μm, s) and larger (>3 μm, L) size fractions. All correlation tests conducted are shown in S4 Fig.

Within each size fraction, the variations in POC and PN concentrations were linked to each other from the surface to the mesopelagic zone, except in the >3 μm size fraction at the DCM and the <3 μm size fraction in the upper mesopelagic zone.

## 4. Discussion

### 4.1 C-fixation patterns and limitations

The present study showed that productivity north of the AzF (106–123 mg C m^-2^ d^-1^) was twice the productivity south of the front (60–77 mg C m^-2^ d^-1^), coinciding with cooler (3–4°C difference between stations C and D), less saline (0.5–0.8 units) and nutrient-enriched waters north of the AzF. These first productivity measurements in the area correspond to the cross-frontal difference in gross primary production (GPP) modeled in a past study for August 1997 (73 and 108 mg C m^-2^ d^-1^, respectively south and north of the AzF; [8]). Macedo *et al*. [8] based their estimates on (i) phytoplankton biomass and ability to use low levels of nitrate (K_S_ = 0.5 μM), (ii) the vertical profiles of nitrate, and (iii) water temperature. Since the Chl fluorescence, nutrient and temperature profiles obtained in the present study are similar to those reported by Macedo *et al*. [8], we can assume that similar conditions of nitrate limitation prevailed. The GPP profiles estimated by Macedo *et al*. [8] match the C-fixation rates we measured in 18MW waters (station A), with peaks of productivity between 40m and 110m indicating strong nitrate limitation. However, the preceding study estimated that the highest GPP would be close to the DCM north of the AzF, while the present study measured the highest C-fixation rates near the surface (11–14m), decreasing towards the DCM. This confirms that *in situ* Chl fluorescence profiles are poor indicators of productivity maxima in the area [46].

In the present study, most of the C-fixation in the euphotic zone was detected at nitrate and phosphate concentrations in the nanomolar range. Vertical and horizontal advection can be important in supplying nutrients [47]. However, past estimates of the upward vertical nitrate fluxes through the nutricline suggested that they may supply only about 25% of the demand north of the NW-AzC/AzF system, and 14% in the 18MW [8]. N_2_-fixation, atmospheric deposition and horizontal nutrient advection might support the rest of the new production. Geochemical, biological and hydrological measurements at the time of sampling were used to evaluate the contribution of these processes.

### 4.2 Geochemical indicators of N_2_-fixation

Landrum *et al*. [18] reported that N_2_-fixation could account for up to 30% of the δ^15^N composition of suspended PN in the Atlantic Ocean area around 32°N-33°W. In the present study, the natural δ^15^N_PN_ measured in the euphotic zone revealed signatures close to published values for the area [18,48], which were generally lower or similar to δ^15^N_NO3_, a characteristic of regions where N_2_-fixation is important [49]. Low δ^15^N_PN_ values may however also result from metabolic transformations [48]. Further evidence for the presence of N_2_-fixation was obtained here from the δ^15^N_NO3_ values <3.5‰ observed down to 300–350m south of the AzF and in the DCM at station D. Such signals are generally attributed to an input of isotopically light ‘new’ nitrate originating from N_2_-fixation, followed by PN remineralisation and nitrification (e.g. [21]). Mixing of any deep water with surface waters would indeed supply nitrate with a higher δ^15^N signature close to 5‰, as reported worldwide [17], and observed at 500m and 1900m depths in station D. Nitrate uptake would then lead to ^15^N enrichment, as observed at station D above 100m, which appeared to benefit from an increased upward flux of ‘heavier’ deep nitrate. At this station, the δ^18^O was strongly negatively correlated with nitrate concentrations, confirming nitrate assimilation [40].

Low δ^15^N_NO3_ could also result from isotopic fractionation during incomplete ammonium oxidation [50]. However, no ammonium accumulation was observed in this study south of the AzF (S3E Fig), invalidating the possibility that low δ^15^N_NO3_ resulted from incomplete ammonium utilization in this area, contrary to station D at 100–125m. Examination of the δ^18^O_NO3_ and δ^15^N_NO3_ covariation revealed that nitrate assimilation used a mixture of deep and ‘new’ (remineralised fixed N_2_) nitrate at all stations. Nitrate in the present study indeed presented intermediate δ^15^N signatures compared to the signals expected from the assimilation of only ‘new’, or only deep, nitrate (Fig 5C). Station C also presented the lowest δ^15^N_NO3_ at 125–250m depths, indicating that N_2_-fixation may be the most important N-source near the AzF.

New N from N_2_-fixation was thus probably incorporated into PN, which became remineralised-nitrified in the subsurface waters, where it added to the pool of upwelled deep nitrate. A mixed layer deepening due to wind stress, and the effect of internal waves can make these subsurface waters with their isotopically light nitrate available to phytoplankton. Atmospheric deposition in the North Atlantic may also explain the presence of isotopically light nitrate at the surface (δ^15^N of -14‰ to 5‰, [51]). However, this N flux is likely small relative to biological N_2_-fixation rates in the oligotrophic North Atlantic [5].

### 4.3 N_2_-fixation and UCYN abundance

Direct measurements of net N_2_-fixation during this study confirmed that the eastern NAST basin close to the Mid-Atlantic Ridge was an area of significant N_2_-fixation activity in the upper 200m of the water column (S5 Table). Although values were two orders of magnitude higher than reported by past studies in the area in spring and autumn [4,32,52], they matched recent rates measured in summer in the western NAST (Pangaea database, [53]), and north of the NW-AzF in a similar hydrological setting [30] (S1G and S1H Fig). Higher rates detected in our study might result from the applied ^15^N_2_ “dissolution method”, which was shown to be more sensitive to the activity of unicellular diazotrophs [25] than the “bubble-addition method” used in previous studies [30]. However, since the temperature in our on-deck incubations (24°C) was 6–9°C higher than the *in situ* temperatures at depths below 48m (15–18°C at DCM-200m), it is possible that C- and N_2_-fixation rates below this depth were over-estimated. North of the AzF, the UCYN cells labeled by Nitro821 in this study were as abundant as the cells detected using the UcynA-732 probe, which targeted only UCYN-A phylotypes amplified from the North Atlantic, in September 2006 at a nearby location [30,33]. Picoplanktonic cells (0.7–1.5 μm) dominated the UCYN population at 99.9% over the whole DIAPICNA transect, with cellular abundances (2–54 cells mL^-1^ at 11–217m) in the 1–140 cells mL^-1^ range the most frequently detected with Nitro821 in Pacific and Mediterranean waters [35,36,37]. In particular, 4.9–7.3 UCYN cells mL^-1^ were detected in surface waters of the most oligotrophic stations south of the AzF, where N_2_-fixation rates of 0.3–0.8 μmol N m^-3^ d^-1^ were simultaneously measured. These values are in the range of rates and cellular abundances measured in past studies in summer 2008 in the Western Mediterranean basin, where N_2_-fixation was estimated to contribute significantly to new primary production [37,54].

Although the only picoplanktonic UCYN known to date belong to group A, with UCYN-B and C being >2–3 μm [30,33,38,55,56], the whole range of cellular shapes, sizes and physiologies is not well known, since only few UCYN representatives have been isolated and cultivated. UCYN diversity is unknown in the study area and the Nitro821 probe targets all UCYN groups. Consequently, we cannot exclude that new free-living picoplanktonic photoautotrophic (as opposed to UCYN-A) members from other groups were detected. Contrary to Krupke *et al*. [30] who reported UCYN-A associations with *Haptophyta* and probably *Alveolata* (mostly >50 m depth), most of the UCYN detected in the present study were free-living in the <3 μm size fraction. Although we cautiously performed gentle filtration, we cannot exclude the possibility that UCYN may have dissociated from their partner cells during size-fractionation, as observed in other studies using a similar filtration protocol [33]. Nevertheless, other UCYN, as well as *Trichodesmium* and non-cyanobacteria prokaryotes, such as particle-associated Gammaproteobacteria, may have contributed to N_2_-fixation [34,57,58]. This might be particularly true at the southern stations A and B, presenting low UCYN cell abundances approaching zero in the DCM, despite the detected significant N_2_-fixation rates. However, *Trichodesmium* and Gammaproteobacteria activities might be restricted to warmer surface waters [31,59]. Even though our protocol was not designed for quantitative *Trichodesmium* sampling, we would have been able to observe trichomes on the >10μm filters, if they would have been present in the sampled water masses. However, trichomes were absent, except for one trichome detected in deep waters at station E, which was probably not active, given the low temperatures measured at this depth.

### 4.4 Factors influencing UCYN abundance, C- and N_2_-fixation

It has been previously reported for the North Atlantic that the surface distribution of diazotrophs followed that of picoplanktonic species, and that multiple abiotic factors influenced their abundance and activity (nutrients, trace metals, O_2_, temperature, [52]). In the present study, the variations in the abundance of UCYN in the smaller size fraction (<3 μm) at the surface (11–16m) were linked to salinity variations, with higher UCYN concentrations in the saltier waters south of the AzF, where the lowest surface C-fixation and the only surface N_2_-fixation rates of the transect were detected. This could be an indication that UCYN may have performed a significant part of the N_2_-fixation observed in surface waters south of the AzF.

The highest UCYN counts were however found 45–200m deep, north of the AzF, in 14.5–20.5°C, oxygenated and nitrate-enriched waters (1–6 μM) with phosphate concentrations of 8–207 nM. Increased UCYN abundance in nutrient-enriched waters has already been observed in earlier studies (e.g. [60,61]). Amendments of 1 μM nitrate alone, or in combination with 200 nM phosphate have also been found to induce UCYN-A *nifH* transcript increases in tropical Atlantic waters [62]. Our results contradict previous conclusions for the North Atlantic regarding the restriction of UCYN to warm waters (>18°C) with sub-micromolar nitrate concentrations [30,31]. However, they are supported by the presence of active UCYN in 14.5–19.0°C Pacific open ocean and North Atlantic shelf waters [61,63], and even in 2.5°C cold waters in the area between the North and Baltic Seas [64]. In the DCM, the picoplanktonic fraction was responsible for half of the total POC production along the transect, except in the least oligrotrophic station D (30%). N_2_-fixation rates sustained on average 45–64% of this production south of the AzF and up to 85% at the northernmost station (using the average 6.2 C/N ratio measured in the picoplanktonic fraction at the DCM along the transect). Since picoplanktonic POC and PN concentrations were directly linked to C-fixation in this size fraction and strongly correlated with total *in situ* Chl fluorescence, we argue that the latter is mainly an indicator of deep picoplanktonic productivity maxima in that area. Chl fluorescence increased with decreasing salinity, which was significantly correlated with lower temperatures that were associated with higher phosphate concentrations. Picoplankton productivity may have therefore been bound to phosphate availability in the DCM, which may also have limited N_2_-fixation and UCYN growth.

In the upper mesopelagic zone, no N_2_-fixation was measured north of the AzF. However, south of the AzF in the larger size fraction, C- and N_2_-fixation might have presented a co-variation, with no relationship to the environmental variables. This might indicate that N_2_-fixation provided new N in association with dark CO_2_-fixation in sinking particles. Future investigations in this area should therefore focus on understanding temporal patterns of N_2_-fixation and obtaining more accurate activity measurements at *in situ* temperature (and hydrostatic pressure).

### 4.5 Impact of the hydrology at the NW-AzC/AzF system

The high shear experienced at the edge of eddies most probably enhances horizontal diapycnal exchange [65], although the mechanisms are still poorly understood. This might increase nutrient supply for new production, particularly in oligotrophic regions where the vertical nitrate flux effectively constrains C-uptake [66]. At the time of sampling, the AzC at 33°W consisted of a large anticyclonic feature sampled at stations B and C, with lowest nutrient concentrations, productivity, biomass, Chl fluorescence and turbidity. An intrusion of northern waters richer in phosphate through the AzF (also observed by Macedo *et al*. [8]) might have induced the slightly increased UCYN counts and N_2_-fixation activity observed in the >3 μm size fraction at station C. The highest UCYN abundances, however, were measured at 45–200m in station E and at 200m in station D. Horizontal diapycnal exchange of subsurface waters from the periphery of the anticyclonic eddy sampled in station E might explain the presence of abundant UCYN at 200m in station D. In addition, surface salinity at station E was between the salinities detected at station D and south of the AzF. This might indicate that waters at station E had been transported from the south and mixed with northern waters (cross-frontal exchange seen in S1A Fig). The transport of *Trichodesmium* filaments from southern waters could explain their presence in northern, colder waters (DCM station E), where they have been rarely reported [31,67].

Enhanced N_2_-fixation rates and UCYN abundances in deep samples at station E may have resulted from the influence of the anticyclonic eddy driving downwelling in its center [14] resulting in slightly higher water temperatures (~1°C) at station E in comparison to station D. Atmospheric P and Fe deposition can also drive increased C-, N_2_-fixation and UCYN abundance, as observed in the tropical North Atlantic (e.g. [62]). It is likely that any influence of a dust deposition event on N_2_-fixation would have been detected in surface waters at both stations D and E, but this was not the case. Moreover, station E was located on the Mid-Atlantic Ridge, on top of a 2300m deep Fe-rich hydrothermal vent field [68]. This Fe source is remote from the surface where N_2_-fixation was detected, but stabilized Fe can be transported over long distances and brought up to shallower water masses [69]. Although we have too little evidence to relate increased N_2_-fixation to a potential influence of hydrothermal Fe, these processes merit further attention in future studies.

## Conclusion

In the present study, marked differences in summer C- and N_2_-fixations were observed across the NW-AzC/AzF system close to the Mid-Atlantic Ridge, presenting contrasted physical-chemical conditions. These first direct measurements of H^13^CO_3_^−^-fixation in the area confirmed previous estimates, with productivity north of the AzF being twice that observed to the South, and nutrient limitation in the euphotic zone over the whole area.

Geochemical measurements revealed the importance of N_2_-fixation in the area, reflected by low particle and nitrate δ^15^N signatures observed down to 200 m depth, resulting from N_2_ incorporation into the particles, followed by remineralisation-nitrification in subsurface waters. South of the front (as well as in an anticyclonic eddy north of the AzF), picoplankton in the DCM performed half of the C-fixation, which was mostly supported by N_2_-fixation. Higher pico-UCYN abundances were however detected in the DCM only in the northern station, where abundances increased down to 200m depth in cool and nutrient-replete waters. At all other stations, the high <3μm N_2_-fixation activity in the DCM was detected in the presence of low UCYN abundance. Other types of prokaryotes might thus contribute to the picoplanktonic diazotrophic activity in the area. Further research is needed to identify the actors of this important activity that sustains C-fixation at the NW-AzC/AzF system.

In upper mesopelagic waters (200–217m depth), C- and N_2_-fixation might have been linked in the >3 μm particles south of the AzF, which could suggest a coupling between N_2_-fixation and dark CO_2_-fixation in sinking particles. This aspect of the dark end of the biological carbon pump should be examined in more detail in future studies.

The intense hydrological dynamics related to the NW-AzC/AzF system appear to influence the biogeochemical processes in the area. The intrusion of southern waters, past the AzF into the nutrient-rich northern waters, associated with eddy-driven downwelling, for instance, corresponded to large increases in UCYN abundances and N_2_-fixation activity in the <3 μm size fraction on the northern side of the front. North-south water mass exchanges as well as diapycnal transfers therefore probably influence the distribution and activities of plankton species in the NW-AzC/AzF area.

## Supporting Information

S1 Fig Real-time AVISO satellite altimetry derived mean geostrophic currents and Sea Surface Height. Day by day sea level anomalies before (A) and at the time of each DIAPICNA station sampling (B, C, D, E, F). Weekly-integrated sea level anomalies during the August 2011 DIAPICNA (G) and September 2006 MSM03/01 VISION cruises (H) indicate that the hydrological setting was similar during both cruises and that the southernmost stations sampled during the VISION cruise were located north of the AzF (see manuscript discussion 4.3). Station locations are marked with dots or stars and the approximate position of the Azores Current-Front system is indicated as a black line.
(PDF)

S2 Fig Depth profiles (m) of physical-chemical parameters. Stations A (day and night), B, C, D and E *in situ* fluorescence (FlECO-AFL), density (Sigma-θ), O_2_ (Oxsol ML/L), salinity, temperature and photo-active radiation (PAR, purple curves) profiles.
(PDF)

S3 Fig Physical-Chemical properties of the water column over the DIAPICNA transect (Y-axis: depth in m). Longitudinal cross-sections of A) Salinity, B) *in situ* chlorophyll fluorescence (mg m^-3^), C) O_2_% saturation and concentrations of D) phosphate in nmol L^-1^, and E) ammonium in μmol L^-1^. The dotted line indicates the position of the AzF.
(PDF)

S4 Fig Spearman correlation coefficient matrixes of the waters’ physico-chemical and biogeochemical properties. Samples collected (A) at the surface (n = 18), (B) above the DCM (n = 18), (C) in the DCM (n = 18) and (D) in the upper mesopelagic (n = 18). The upper right panels show the pairwise scatterplots. A smoothing curve (LOESS) with a span of 0.66 was added for visual interpretation. The lower left panels show the correlation coefficient (Spearman rank), including significant p-values. Histograms of the variables are included in the diagonal. Significant correlations at p<0.001, p<0.01 and p<0.05 are indicated with ***, ** and *, and highlighted in red, orange and yellow, respectively. The numbers at the top, bottom and sides of the multipanel figure are the units of the respective variable.
(PDF)

S1 Table Particulate Nitrogen enrichment for <3 μm and >3 μm particles. Particles collected during the day in the euphotic zone. Corrected (i.e. given the value of natural SD/2) if <Depth 3xSD. Flagged in grey if <0.0908 (highest error from ^15^N_2_ replicates). Flagged in black if N_2_ fixation <propagated error *E*.
(PDF)

S2 Table Particulate organic carbon enrichment for <3 μm and >3 μm particles. Particles collected during the day in the euphotic zone. *E* values represent calibration uncertainties and propagated errors at each step of the calculation.
(PDF)

S3 Table Particulate organic carbon and nitrogen enrichments for the night cast at station 1. Corrected (i.e. given the value of natural SD/2) if <Depth 3xSD. Flagged in grey if <0.0908 (highest error from ^15^N_2_ replicates). Flagged in black if N_2_ fixation <propagated error *E*.
(PDF)

S4 Table Correlations between the physical-chemical and biogeochemical variables. Spearman correlations’ significance at p<0.001, p<0.01 and p<0.05 are shown with ***, ** and *, respectively. O_2_: oxygen saturation %; Temp: potential temperature; Sal: salinity; PAR: % surface photoactive radiation; Chl: in situ Chlorophyll fluorescence. POM, POC, PN: Particulate Organic Matter, Carbon, Nitrogen concentrations; C-Fix, N_2_-Fix: C- and N_2_-fixation and Pico-UCYN abundance are examined for the small (<3 μm, s) and larger (>3 μm, L) size fractions.
(PDF)

S5 Table Comparison of N_2_-fixation volumetric rates measured in the Subtropical Mid-Atlantic between 2006 and 2011. (PDF)

S1 Text Supporting material and methods, results and references. (PDF)
