# Supplementary figures and images for: Importance of N2-Fixation on the Productivity at the North-Western Azores Current/Front System, and the Abundance of Diazotrophic Unicellular Cyanobacteria
Source: PLoS One. 2016 Mar 9;11(3):e0150827. doi: 10.1371/journal.pone.0150827 (PMC4784884; doi:10.1371/journal.pone.0150827)

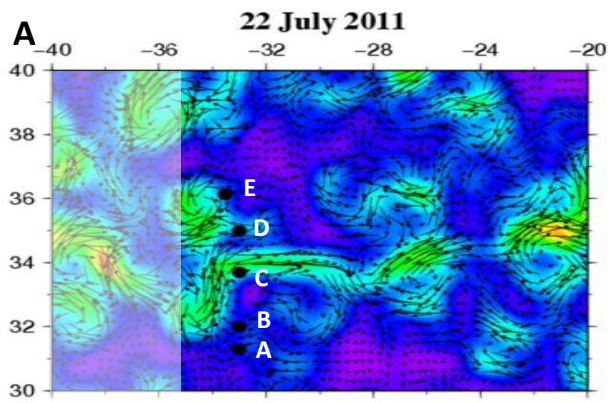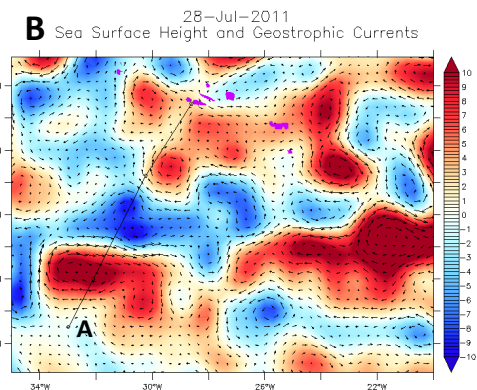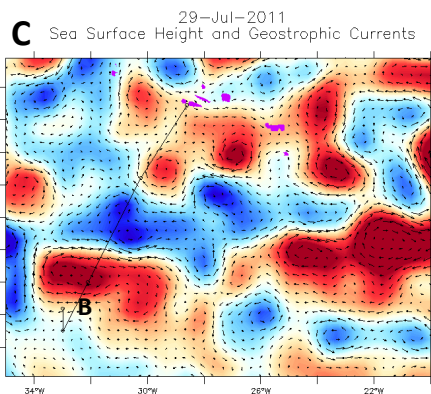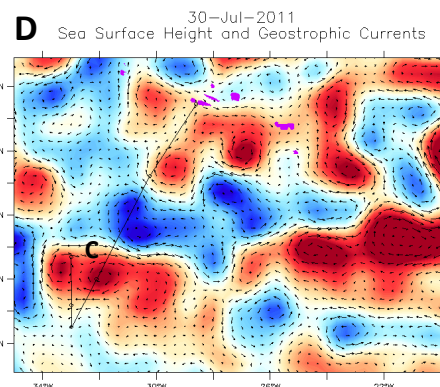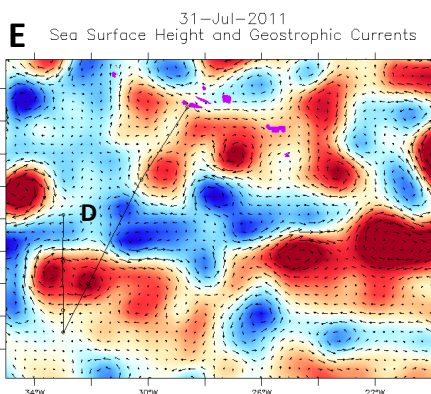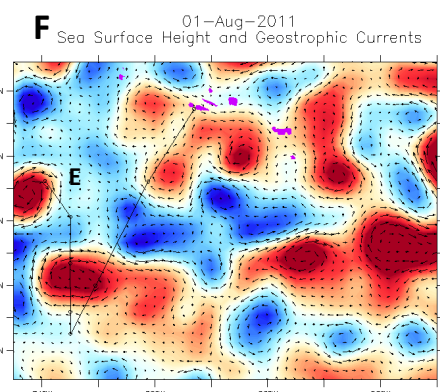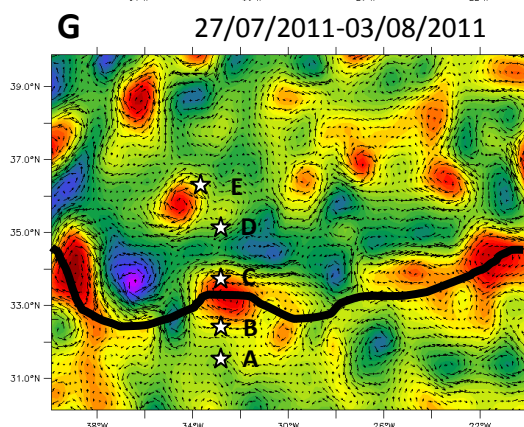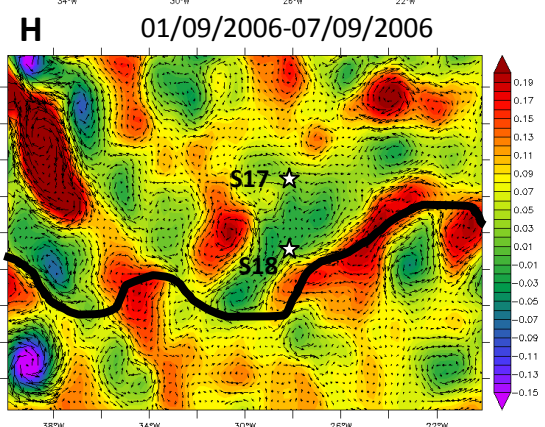

Supplement: S1 Fig — Day by day sea level anomalies before (A) and at the time of each DIAPICNA station sampling (B, C, D, E, F). Weekly-integrated sea level anomalies during the August 2011 DIAPICNA (G) and September 2006 MSM03/01 VISION cruises (H) indicate that the hydrological setting was similar during both cruises and that the southernmost stations sampled during the VISION cruise were located north of the AzF (see manuscript discussion 4.3). Station locations are marked with dots or stars and the approximate position of the Azores Current-Front system is indicated as a black line. (PDF) [file pone.0150827.s001.pdf]

Day

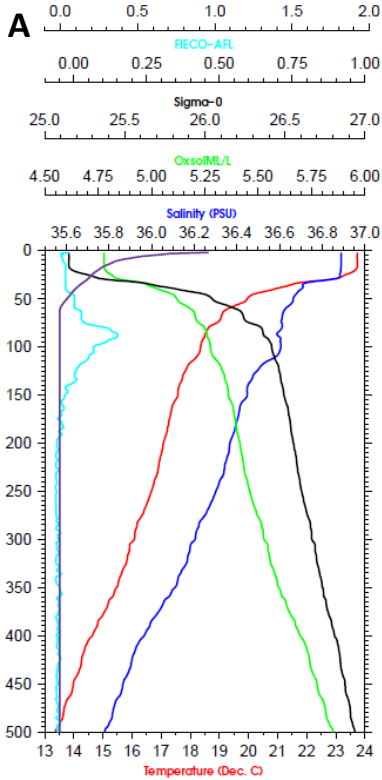

Night

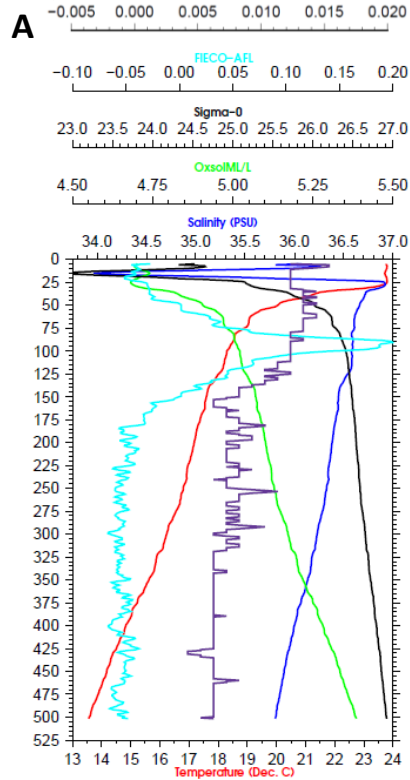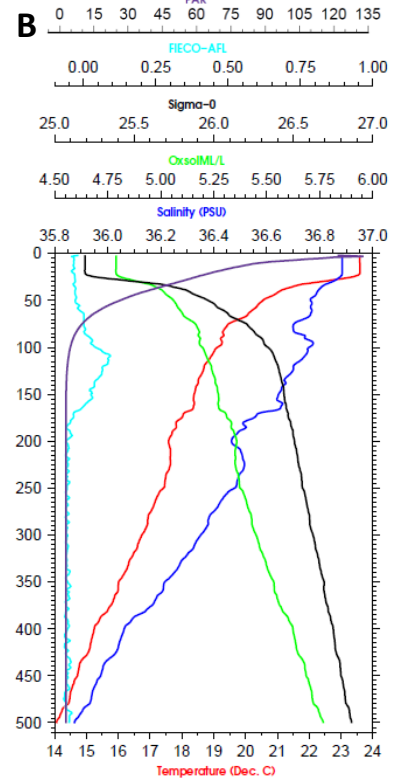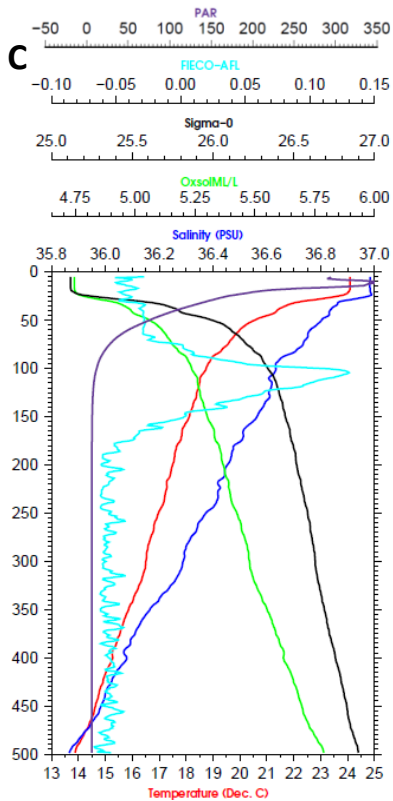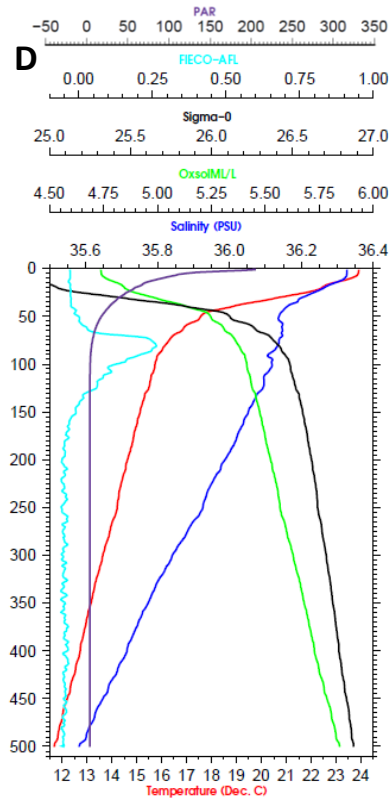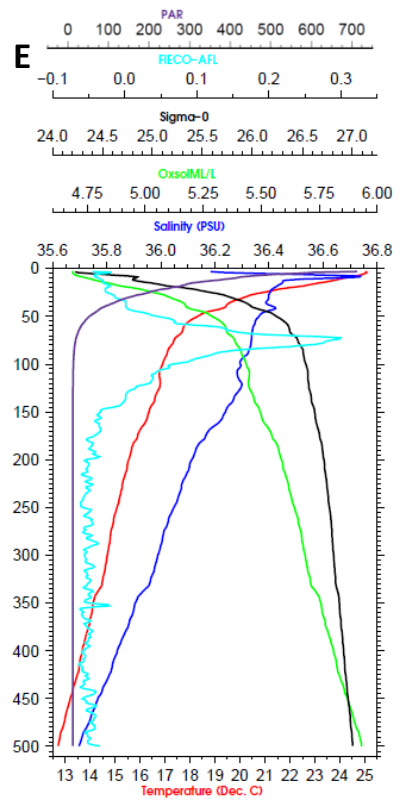

Supplement: S2 Fig — Stations A (day and night), B, C, D and E in situ fluorescence (FlECO-AFL), density (Sigma-θ), O2 (Oxsol ML/L), salinity, temperature and photo-active radiation (PAR, purple curves) profiles. (PDF) [file pone.0150827.s002.pdf]

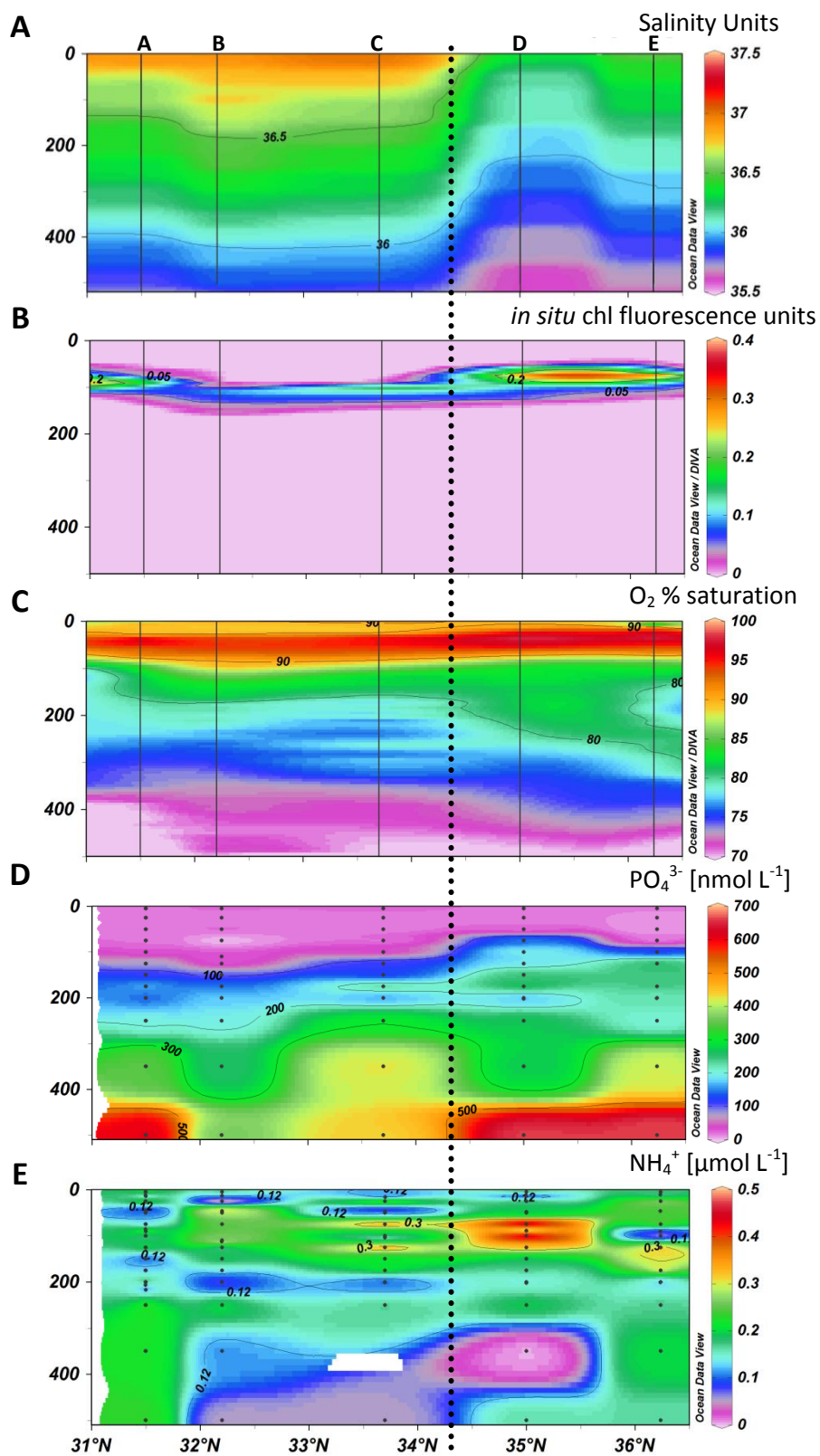

Supplement: S3 Fig — Longitudinal cross-sections of A) Salinity, B) in situ chlorophyll fluorescence (mg m-3), C) O2% saturation and concentrations of D) phosphate in nmol L-1, and E) ammonium in μmol L-1. The dotted line indicates the position of the AzF. (PDF) [file pone.0150827.s003.pdf]

**A**

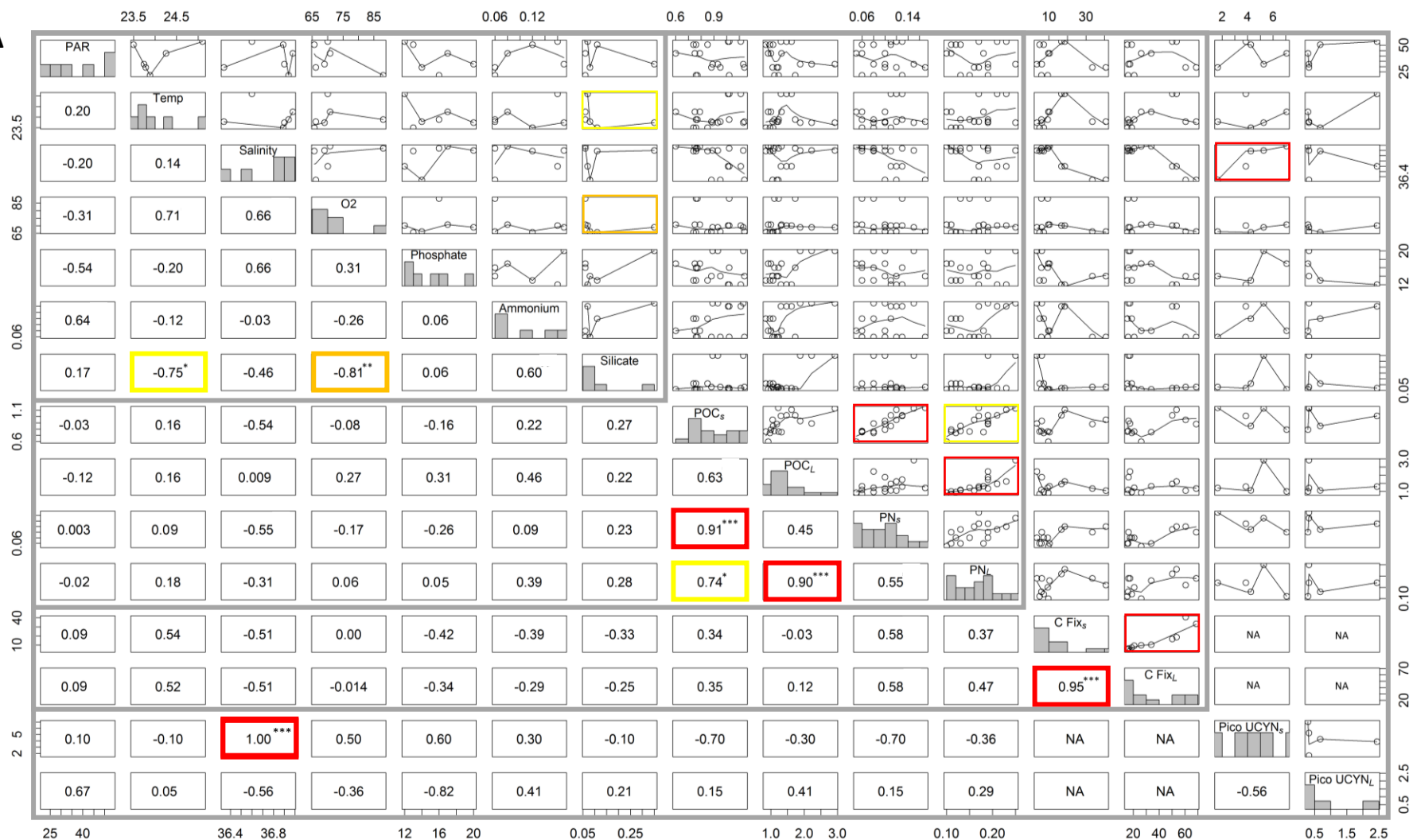

**B**

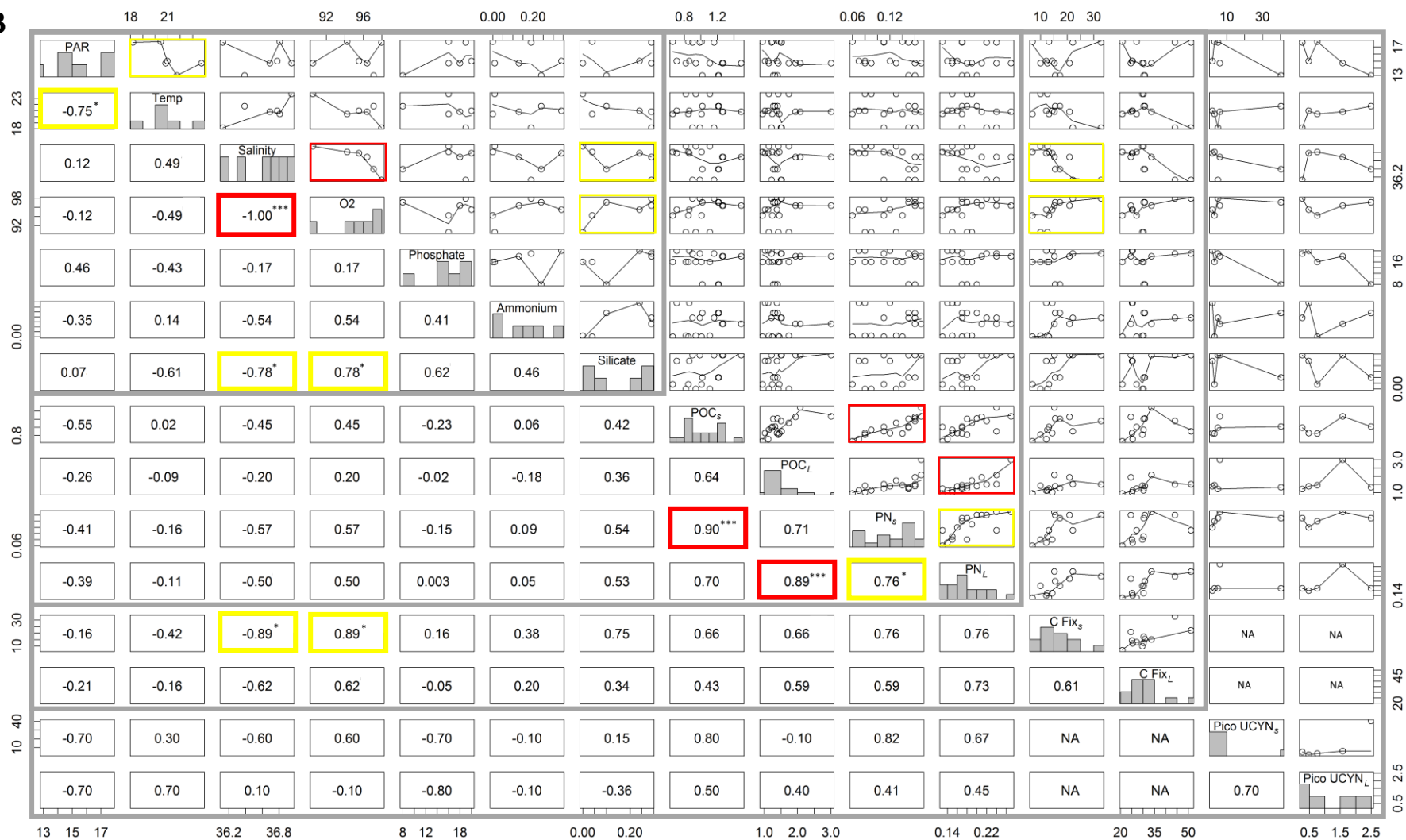

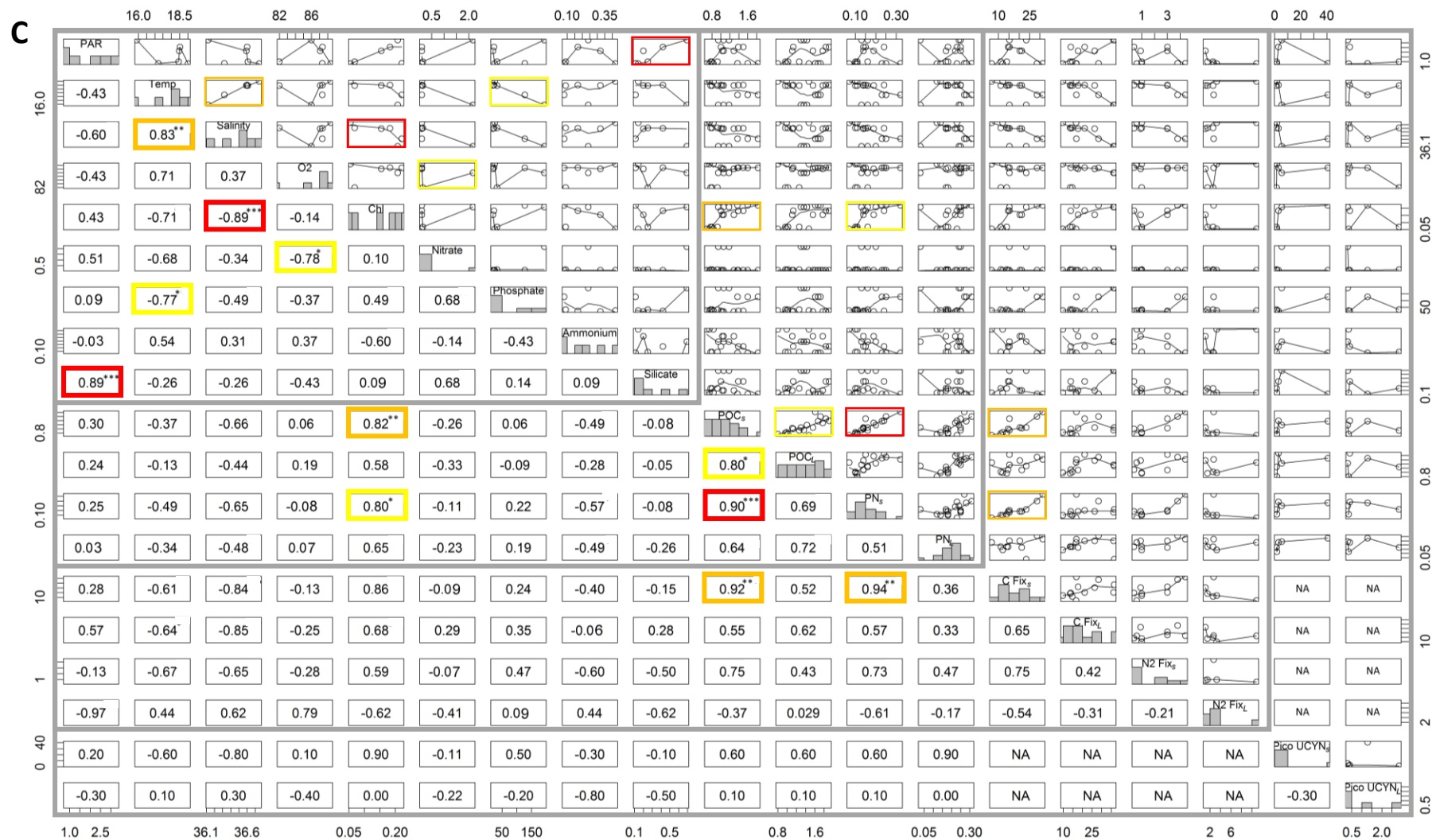

D

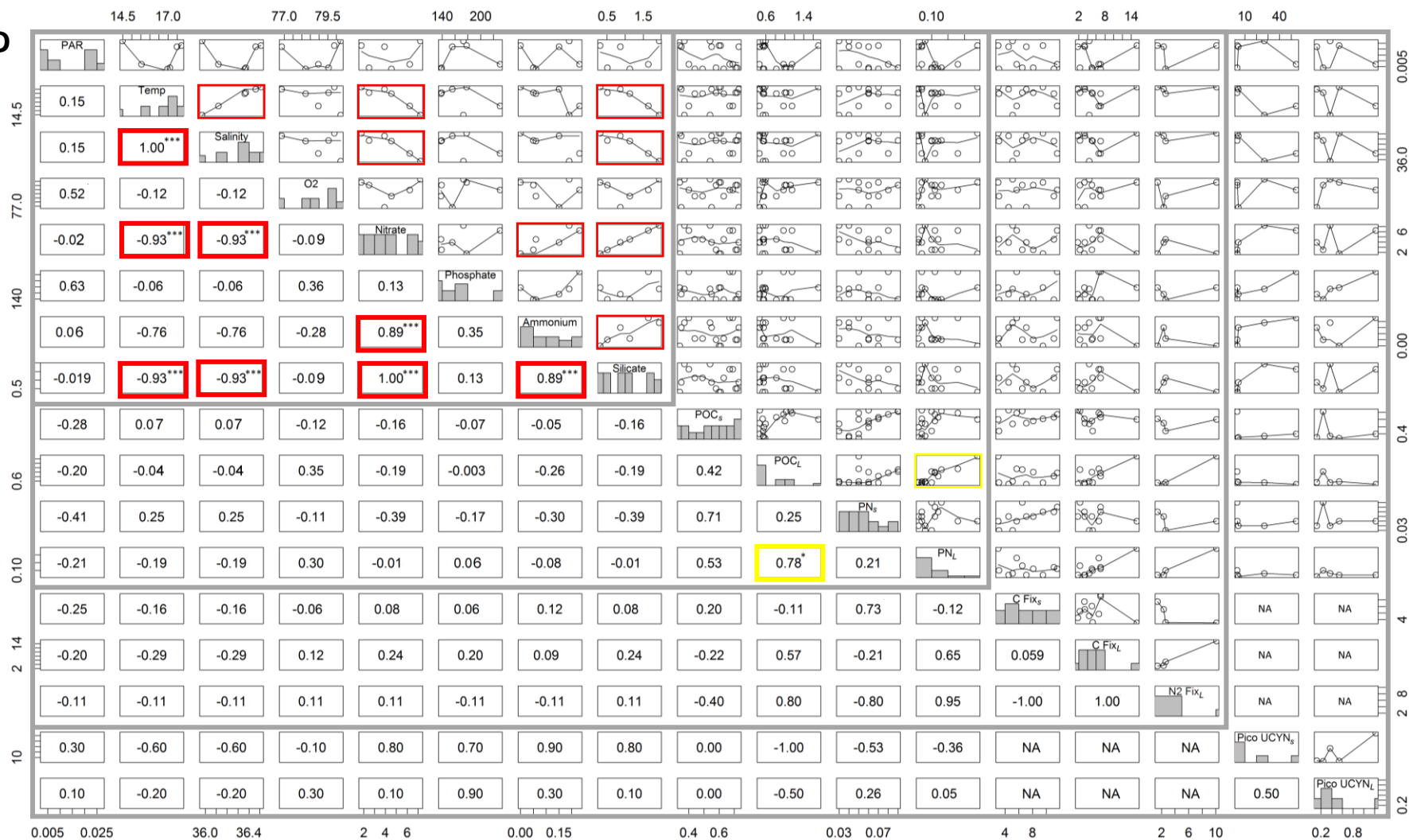

Supplement: S4 Fig — Samples collected (A) at the surface (n = 18), (B) above the DCM (n = 18), (C) in the DCM (n = 18) and (D) in the upper mesopelagic (n = 18). The upper right panels show the pairwise scatterplots. A smoothing curve (LOESS) with a span of 0.66 was added for visual interpretation. The lower left panels show the correlation coefficient (Spearman rank), including significant p-values. Histograms of the variables are included in the diagonal. Significant correlations at p<0.001, p<0.01 and p<0.05 are indicated with ***, ** and *, and highlighted in red, orange and yellow, respectively. The numbers at the top, bottom and sides of the multipanel figure are the units of the respective variable. (PDF) [file pone.0150827.s004.pdf]
