# Supplementary material for: Importance of N2-Fixation on the Productivity at the North-Western Azores Current/Front System, and the Abundance of Diazotrophic Unicellular Cyanobacteria
Source: PLoS One. 2016 Mar 9;11(3):e0150827. doi: 10.1371/journal.pone.0150827 (PMC4784884; doi:10.1371/journal.pone.0150827)
Supplement: S1 Table — Particles collected during the day in the euphotic zone. Corrected (i.e. given the value of natural SD/2) if <Depth 3xSD. Flagged in grey if <0.0908 (highest error from 15N2 replicates). Flagged in black if N2 fixation <propagated error E. (PDF) [file pone.0150827.s005.pdf]

PN <3 µm		t = 0 Natural PN $\delta^{15}N_{PN}$ [‰]	*E*	*Depth 3xSD* [‰]	Atom%	t=24h Incubated PN $\delta^{15}N_{PN}$ [‰]	*E*	Concentration [µmol/4.5L]	*E*	C:N ratio	PN Enrichment $\delta^{15}N$ [‰]	$^{15}N^{A}$ Atom%	*E*	Corrected Atom%	t=24h Dissolved N_2 ^{15}N Atom%	*SD*	$^{final}A_{substrate} - {}^{t=0}A_{particle}$ $^{15}N^{B}$ Atom%	*E*	N_2 fixation [nmol/L]	N_2 fixation rate [µmol N/m^3/d]	*E*
Station A Day	12m	1.54	*0.91*	*3.40*	*0.0012*	4.49	*1.99*	0.46	*0.12*	9.16	2.95	0.0011	*0.0006*	0.0002	0.8348	*0.0284*	0.4680	*0.0284*	0.045	0.045	0.128
						2.44	*0.63*	0.36	*0.05*	10.92	0.90	0.0003	*0.0006*	0.0002	0.9068	*0.0075*	0.5399	*0.0076*	0.031	0.031	0.088
	45m	1.23	*0.79*	*13.85*	*0.0051*	11.09	*1.33*	0.70	*0.12*	8.08	9.86	0.0036	*0.0024*	0.0008	0.9650	*0.0094*	0.5982	*0.0095*	0.220	0.220	0.624
						9.23	*0.36*	0.77	*0.05*	8.63	8.00	0.0029	*0.0024*	0.0008	0.7808	*0.0028*	0.4141	*0.0033*	0.351	0.351	0.992
	86m	3.14	*0.57*	*2.69*	*0.0010*	6.82	*1.46*	0.64	*0.12*	7.06	3.68	0.0013	*0.0005*	0.0013	0.8245	*0.0704*	0.4570	*0.0704*	0.418	0.413	0.174
						39.14	*0.69*	0.69	*0.05*	9.75	35.99	0.0131	*0.0005*	0.0131	1.0292	*0.0158*	0.6618	*0.0158*	3.058	3.027	0.268
	207m	2.17	*1.53*	*13.14*	*0.0048*	13.51	*2.36*	0.38	*0.12*	8.45	11.34	0.0041	*0.0023*	0.0008	0.9130	*0.0041*	0.5459	*0.0044*	0.125	0.119	0.340
						9.92	*0.82*	0.27	*0.05*	9.82	7.75	0.0028	*0.0023*	0.0008	0.9565	*0.0174*	0.5894	*0.0175*	0.081	0.077	0.218
Station B	13m	0.16	*1.25*	*3.40*	*0.0012*	4.13	*2.62*	0.34	*0.12*	9.71	3.97	0.0014	*0.0006*	0.0014	0.7879	*0.0024*	0.4215	*0.0025*	0.261	0.246	0.132
						1.13	*0.79*	0.27	*0.05*	12.61	0.97	0.0004	*0.0006*	0.0002	0.7405	*0.0036*	0.3741	*0.0036*	0.033	0.031	0.089
	46m	-0.20	*1.11*	*13.85*	*0.0051*	3.91	*2.83*	0.31	*0.12*	10.08	4.11	0.0015	*0.0024*	0.0008	0.5232	*0.0012*	0.1570	*0.0020*	0.376	0.365	1.040
						3.39	*0.61*	0.38	*0.05*	10.21	3.60	0.0013	*0.0024*	0.0008	0.5797	*0.0019*	0.2135	*0.0026*	0.331	0.321	0.909
	112m	2.24	*0.86*	*2.69*	*0.0010*	6.68	*2.04*	0.45	*0.12*	8.36	4.44	0.0016	*0.0005*	0.0016	0.5162	*0.0633*	0.1491	*0.0633*	1.083	1.039	0.601
						7.15	*0.75*	0.29	*0.05*	10.40	4.91	0.0018	*0.0005*	0.0018	0.5472	*0.0032*	0.1801	*0.0032*	0.654	0.628	0.199
	200m	13.97	*3.08*	*13.14*	*0.0048*	11.70	*2.82*	0.32	*0.12*	8.73	-2.28	-0.0008	*0.0023*	0.0008	0.7667	*0.0167*	0.3953	*0.0168*	0.142	0.137	0.390
						12.50	*0.99*	0.21	*0.05*	13.09	-1.47	-0.0005	*0.0023*	0.0008	0.4887	*0.0035*	0.1173	*0.0039*	0.314	0.301	0.855
Station C	16m	2.73	*1.40*	*3.40*	*0.0012*	4.86	*3.55*	0.24	*0.12*	11.03	2.13	0.0008	*0.0006*	0.0002	0.4070	*0.0017*	0.0397	*0.0017*	0.284	0.263	0.756
						4.91	*0.76*	0.29	*0.05*	11.66	2.18	0.0008	*0.0006*	0.0002	0.6910	*0.0040*	0.3237	*0.0040*	0.041	0.038	0.108
	47m	11.01	*0.95*	*13.85*	*0.0051*	3.52	*1.88*	0.48	*0.12*	9.30	-7.49	-0.0027	*0.0024*	0.0008	0.4441	*0.0132*	0.0738	*0.0133*	1.235	1.170	3.328
						5.83	*0.76*	0.29	*0.05*	10.17	-5.18	-0.0019	*0.0024*	0.0008	0.3859	*0.0006*	0.0156	*0.0018*	3.492	3.308	9.384
	104m	2.24	*0.86*	*2.69*	*0.0010*	-0.88	*1.99*	0.46	*0.12*	7.60	-3.12	-0.0011	*0.0005*	0.0002	0.5163	*0.0011*	0.1492	*0.0012*	0.112	0.106	0.300
						6.79	*0.50*	0.49	*0.05*	7.33	4.55	0.0017	*0.0005*	0.0017	0.5383	*0.0109*	0.1712	*0.0109*	1.065	1.005	0.308
	201m	7.11	*3.10*	*13.14*	*0.0048*	7.50	*3.51*	0.25	*0.12*	9.91	0.38	0.0001	*0.0023*	0.0008	0.4836	*0.0046*	0.1147	*0.0049*	0.386	0.386	1.106
						14.11	*0.86*	0.25	*0.05*	12.89	7.00	0.0026	*0.0023*	0.0008	0.3978	*0.0010*	0.0289	*0.0019*	1.563	1.563	4.434
Station D	14m	2.57	*0.76*	*3.40*	*0.0012*	4.27	*1.73*	0.53	*0.12*	8.03	1.70	0.0006	*0.0006*	0.0002	0.4658	*0.0005*	0.0986	*0.0007*	0.248	0.233	0.660
						1.86	*0.53*	0.45	*0.05*	8.79	-0.71	-0.0003	*0.0006*	0.0002	0.3936	*0.0017*	0.0264	*0.0017*	0.785	0.736	2.084
	48m	5.42	*0.90*	*13.85*	*0.0051*	5.68	*1.33*	0.70	*0.12*	7.46	0.26	0.0001	*0.0024*	0.0008	0.5368	*0.0017*	0.1685	*0.0024*	0.779	0.738	2.091
						2.89	*0.49*	0.49	*0.05*	8.15	-2.53	-0.0009	*0.0024*	0.0008	0.4925	*0.0003*	0.1242	*0.0017*	0.749	0.709	2.007
	89m	0.70	*0.66*	*2.69*	*0.0010*	2.32	*1.33*	0.70	*0.12*	6.75	1.63	0.0006	*0.0005*	0.0002	0.4593	*0.0013*	0.0927	*0.0014*	0.276	0.260	0.737
						1.49	*0.42*	0.58	*0.05*	7.19	0.80	0.0003	*0.0005*	0.0002	0.4147	*0.0002*	0.0481	*0.0004*	0.443	0.417	1.181
	202m	11.74	*3.41*	*13.14*	*0.0048*	8.21	*1.20*	0.15	*0.05*	15.83	-3.52	-0.0013	*0.0023*	0.0008	0.4344	*0.0017*	0.0638	*0.0023*	0.428	0.402	1.147
Station E	11m	0.74	*1.02*	*3.40*	*0.0012*	6.71	*1.73*	0.53	*0.12*	9.39	5.96	0.0022	*0.0006*	0.0022	0.3975	*0.0022*	0.0309	*0.0022*	8.350	7.937	2.840
						3.01	*0.50*	0.48	*0.05*	9.51	2.26	0.0008	*0.0006*	0.0002	0.4957	*0.0201*	0.1292	*0.0201*	0.171	0.163	0.462
	46m	-0.30	*0.90*	*13.85*	*0.0051*	4.49	*1.42*	0.65	*0.12*	8.35	4.80	0.0018	*0.0024*	0.0008	0.4437	*0.0092*	0.0775	*0.0094*	1.579	1.526	4.329
						5.54	*0.37*	0.70	*0.05*	7.82	5.84	0.0021	*0.0024*	0.0008	0.4276	*0.0237*	0.0614	*0.0238*	2.140	2.068	5.907
	90m	3.13	*0.57*	*2.69*	*0.0010*	8.14	*0.68*	1.43	*0.12*	5.75	5.01	0.0018	*0.0005*	0.0018	0.4974	*0.0020*	0.1300	*0.0021*	4.493	4.447	1.189
						7.32	*0.27*	1.12	*0.05*	6.40	4.20	0.0015	*0.0005*	0.0015	0.4669	*0.0163*	0.0995	*0.0163*	3.845	3.805	1.323
	201m	4.77	*2.76*	*13.14*	*0.0048*	11.23	*2.64*	0.34	*0.12*	8.79	6.45	0.0024	*0.0023*	0.0008	0.4303	*0.0206*	0.0623	*0.0207*	0.973	0.959	2.753
						3.24	*0.87*	0.24	*0.05*	13.05	-1.53	-0.0006	*0.0023*	0.0008	0.4369	*0.0204*	0.0689	*0.0205*	0.618	0.610	1.739

PN >3µm		t = 0 Natural PN				t=24h Incubated PN					PN Enrichment				t=24h Dissolved N_2		$^{final}A_{substrate} - ^{t=0}A_{particle}$		N_2 fixation	N_2 fixation rate	
		$\delta^{15}N$		*Depth 3xSD*		$\delta^{15}N$		Concentration		C:N	$\delta^{15}N$	^{15}N [A]		**Corrected**	^{15}N		^{15}N [B]				
		[‰]	*E*	[‰]	Atom%	[‰]	*E*	[µmol/4.5L]	*E*	ratio	[‰]	Atom%	*E*	Atom%	Atom%	*E*	Atom%	*E*	[nmol/L]	[µmol N/m^3/d]	*E*
Station A Day	12m	0.23	*1.53*	*2.44*	*0.0009*	1.87	*0.46*	0.87	*0.12*	9.67	1.63	0.0006	0.0004	0.0001	0.8348	*0.0284*	0.4684	*0.0284*	0.062	0.062	*0.044*
						1.41	*0.25*	0.84	*0.10*	11.81	1.18	0.0004	0.0004	0.0001	0.9068	*0.0075*	0.5404	*0.0076*	0.052	0.052	*0.051*
	45m	0.63	*1.38*	*4.62*	*0.0017*	2.07	*0.43*	0.94	*0.12*	9.26	1.43	0.0005	0.0008	0.0003	0.9650	*0.0094*	0.5984	*0.0094*	0.098	0.098	*0.150*
						4.57	*0.21*	1.09	*0.10*	8.54	3.94	0.0014	0.0008	0.0003	0.7808	*0.0028*	0.4143	*0.0029*	0.166	0.166	*0.093*
	86m	2.49	*1.63*	*1.74*	*0.0006*	3.51	*0.50*	0.81	*0.12*	6.22	1.02	0.0004	0.0003	0.0001	0.8245	*0.0704*	0.4573	*0.0704*	0.042	0.042	*0.034*
						4.12	*0.21*	1.10	*0.10*	7.50	1.63	0.0006	0.0003	0.0006	1.0292	*0.0158*	0.6620	*0.0158*	0.220	0.218	*0.112*
	207m	2.80	*3.98*	*6.92*	*0.0025*	4.24	*0.71*	0.60	*0.12*	8.40	1.44	0.0005	0.0012	0.0004	0.9130	*0.0041*	0.5456	*0.0042*	0.103	0.099	*0.225*
						5.45	*0.47*	0.49	*0.10*	9.12	2.65	0.0010	0.0012	0.0004	0.9565	*0.0174*	0.5892	*0.0174*	0.079	0.075	*0.094*
Station B	13m	1.08	*0.74*	*2.44*	*0.0009*	2.80	*0.39*	0.54	*0.10*	8.01	1.72	0.0006	0.0004	0.0001	0.7879	*0.0024*	0.4212	*0.0025*	0.043	0.040	*0.028*
						2.83	*0.43*	0.50	*0.10*	7.69	1.75	0.0006	0.0004	0.0001	0.7405	*0.0036*	0.3738	*0.0036*	0.044	0.042	*0.028*
	46m	3.81	*0.58*	*4.62*	*0.0017*	4.24	*0.36*	0.61	*0.10*	8.20	0.42	0.0002	0.0008	0.0003	0.5232	*0.0012*	0.1556	*0.0013*	0.246	0.239	*1.227*
						1.40	*0.32*	0.65	*0.10*	7.05	-2.41	-0.0009	0.0008	0.0003	0.5797	*0.0019*	0.2120	*0.0020*	0.192	0.186	*0.171*
	112m	3.74	*0.81*	*1.74*	*0.0006*	13.91	*0.51*	0.63	*0.10*	7.12	10.17	0.0037	0.0003	0.0037	0.5162	*0.0633*	0.1485	*0.0633*	3.489	3.349	*1.549*
						28.19	*0.52*	1.03	*0.10*	6.45	24.45	0.0089	0.0003	0.0089	0.5472	*0.0032*	0.1796	*0.0032*	11.369	10.914	*1.145*
	200m	-0.31	*1.50*	*6.92*	*0.0025*	21.51	*1.31*	0.32	*0.10*	7.41	21.82	0.0080	0.0012	0.0080	0.7667	*0.0167*	0.4005	*0.0167*	1.423	1.366	*0.477*
						14.49	*0.31*	1.07	*0.10*	7.20	14.80	0.0054	0.0012	0.0054	0.4887	*0.0035*	0.1225	*0.0036*	10.502	10.082	*2.439*
Station C	16m	1.01	*0.83*	*2.44*	*0.0009*	3.87	*0.49*	0.44	*0.10*	9.37	2.86	0.0010	0.0004	0.0010	0.4070	*0.0017*	0.0403	*0.0017*	2.554	2.365	*1.097*
						4.81	*0.28*	0.81	*0.10*	7.25	3.79	0.0014	0.0004	0.0014	0.6910	*0.0040*	0.3243	*0.0040*	0.775	0.718	*0.235*
	47m	4.26	*0.55*	*4.62*	*0.0017*	6.04	*0.39*	0.60	*0.10*	9.31	1.79	0.0007	0.0008	0.0003	0.4441	*0.0132*	0.0763	*0.0133*	0.491	0.465	*0.578*
						5.02	*0.39*	0.59	*0.10*	7.22	0.77	0.0003	0.0008	0.0003	0.3859	*0.0006*	0.0180	*0.0008*	2.044	1.936	*5.516*
	104m	2.31	*0.52*	*1.74*	*0.0006*	8.49	*0.31*	0.83	*0.10*	5.53	6.17	0.0023	0.0003	0.0023	0.5163	*0.0011*	0.1492	*0.0011*	2.780	2.625	*0.473*
						6.55	*0.30*	0.81	*0.10*	5.33	4.23	0.0015	0.0003	0.0015	0.5383	*0.0109*	0.1712	*0.0109*	1.631	1.541	*0.367*
	201m	5.82	*1.98*	*6.92*	*0.0025*	16.26	*1.13*	0.31	*0.10*	6.68	10.45	0.0038	0.0012	0.0038	0.4836	*0.0046*	0.1152	*0.0047*	2.267	2.267	*1.030*
						19.48	*1.25*	0.31	*0.10*	6.80	13.66	0.0050	0.0012	0.0050	0.3978	*0.0010*	0.0294	*0.0013*	11.780	11.780	*4.760*
Station D	14m	2.39	*0.52*	*2.44*	*0.0009*	3.54	*0.30*	0.73	*0.10*	6.48	1.15	0.0004	0.0004	0.0001	0.4658	*0.0005*	0.0986	*0.0006*	0.244	0.228	*0.230*
						2.59	*0.25*	0.86	*0.10*	6.13	0.21	0.0001	0.0004	0.0001	0.3936	*0.0017*	0.0265	*0.0017*	1.075	1.008	*5.642*
	48m	3.21	*0.54*	*4.62*	*0.0017*	9.05	*0.27*	0.97	*0.10*	6.91	5.84	0.0021	0.0008	0.0021	0.5368	*0.0017*	0.1693	*0.0018*	2.734	2.590	*1.002*
						6.35	*0.22*	1.09	*0.10*	6.23	3.14	0.0011	0.0008	0.0003	0.4925	*0.0003*	0.1250	*0.0006*	0.547	0.518	*0.363*
	89m	2.19	*0.37*	*1.74*	*0.0006*	-0.98	*1.35*	0.15	*0.10*	7.73	-3.16	-0.0012	0.0003	0.0001	0.4593	*0.0013*	0.0922	*0.0014*	0.037	0.035	*0.026*
						3.50	*0.20*	1.11	*0.10*	5.51	1.31	0.0005	0.0003	0.0001	0.4147	*0.0002*	0.0476	*0.0003*	0.550	0.518	*0.327*
	202m	2.72	*1.33*	*6.92*	*0.0025*	8.67	*0.74*	0.35	*0.10*	7.65	5.95	0.0022	0.0012	0.0004	0.4344	*0.0017*	0.0671	*0.0019*	0.486	0.457	*0.284*
Station E	11m	1.79	*0.54*	*2.44*	*0.0009*	3.70	*0.21*	1.04	*0.10*	6.95	1.91	0.0007	0.0004	0.0001	0.3975	*0.0022*	0.0306	*0.0022*	1.130	1.074	*0.660*
						2.66	*0.23*	0.94	*0.10*	7.03	0.87	0.0003	0.0004	0.0001	0.4957	*0.0201*	0.1288	*0.0201*	0.241	0.229	*0.306*
	46m	1.40	*0.52*	*4.62*	*0.0017*	4.47	*0.27*	0.83	*0.10*	6.91	3.08	0.0011	0.0008	0.0003	0.4437	*0.0092*	0.0769	*0.0092*	0.673	0.650	*0.474*
						3.58	*0.24*	0.92	*0.10*	6.86	2.18	0.0008	0.0008	0.0003	0.4276	*0.0237*	0.0608	*0.0237*	0.944	0.912	*0.982*
	90m	3.08	*0.32*	*1.74*	*0.0006*	6.70	*0.21*	1.19	*0.10*	6.06	3.62	0.0013	0.0003	0.0013	0.4974	*0.0020*	0.1300	*0.0021*	2.693	2.665	*0.647*
						4.45	*0.17*	1.37	*0.10*	5.63	1.37	0.0005	0.0003	0.0001	0.4669	*0.0163*	0.0995	*0.0163*	0.325	0.322	*0.201*
	201m	4.29	*1.57*	*6.92*	*0.0025*	8.08	*0.50*	0.50	*0.10*	8.67	3.79	0.0014	0.0012	0.0004	0.4303	*0.0206*	0.0624	*0.0206*	0.757	0.746	*0.706*
						11.07	*0.35*	0.81	*0.10*	6.32	6.78	0.0025	0.0012	0.0025	0.4369	*0.0204*	0.0690	*0.0204*	6.512	6.423	*3.721*
