# Supplementary material for: Importance of N2-Fixation on the Productivity at the North-Western Azores Current/Front System, and the Abundance of Diazotrophic Unicellular Cyanobacteria
Source: PLoS One. 2016 Mar 9;11(3):e0150827. doi: 10.1371/journal.pone.0150827 (PMC4784884; doi:10.1371/journal.pone.0150827)
Supplement: S2 Table — Particles collected during the day in the euphotic zone. E values represent calibration uncertainties and propagated errors at each step of the calculation. (PDF) [file pone.0150827.s006.pdf]

**POC <3 µm**		**t = 0 Natural POC**		**t=24h Incubated POC**			**POC Enrichment**		**t=24h DIC**		**$^{final}A_{substrate} - {}^{t=0}A_{particle}$**						
		^{13}C	*Depth 3xSD*	^{13}C	Concentration		^{13}C		^{13}C		^{13}C		**Uptake C**		**Uptake rate**	**#**	**Uptake C**
		Atom%	Atom%	Atom%	[µmol/4.5L]	*E*	Atom%	*E*	Atom%	*E*	Atom%	*E*	**[nmol/l]**	*E*	**[µmol C/m^3/d]**	**meters**	**[mmol C/m²/d]**
Station A Day	12m	1.0821	*0.0031*	1.1354	4.19	*0.71*	0.0533	*0.0044*	9.1132	*0.0342*	8.0312	*0.0344*	6.2	*1.2*	**4.9**	25	
				1.1143	3.98	*0.63*	0.0323	*0.0044*	8.9680	*0.0158*	7.8859	*0.0161*	3.6	*0.8*			
	45m	1.0826	*0.0016*	1.2140	5.66	*0.71*	0.1314	*0.0022*	9.0213	*0.0051*	7.9388	*0.0053*	20.9	*2.6*	**18.0**	40	
				1.1684	6.67	*0.63*	0.0858	*0.0022*	9.4909	*0.0156*	8.4083	*0.0157*	15.2	*1.5*			
	86m	1.0817	*0.0025*	1.1955	4.50	*0.71*	0.1139	*0.0036*	8.8265	*0.0036*	7.7448	*0.0044*	14.8	*2.3*	**14.9**	77.5	
				1.1628	6.74	*0.63*	0.0812	*0.0036*	8.9838	*0.0016*	7.9021	*0.0030*	15.4	*1.6*			
	207m	1.0799	*0.0040*	1.1585	3.24	*0.71*	0.0787	*0.0057*	9.4700	*0.0081*	8.3901	*0.0091*	6.8	*1.5*	**7.3**	57.5	
				1.1927	2.62	*0.63*	0.1128	*0.0057*	8.8531	*0.0020*	7.7733	*0.0045*	8.5	*2.0*			**2.42**
Station B	13m	1.0814	*0.0031*	1.1629	3.31	*0.71*	0.0815	*0.0044*	8.9326	*0.0031*	7.8512	*0.0044*	7.7	*1.6*	**6.1**	25	
				1.1367	3.41	*0.63*	0.0553	*0.0044*	8.9088	*0.0039*	7.8274	*0.0050*	5.4	*1.0*			
	46m	1.0826	*0.0016*	1.2226	3.17	*0.71*	0.1400	*0.0022*	8.9367	*0.0017*	7.8541	*0.0023*	12.6	*2.7*	**13.3**	52.5	
				1.2190	3.84	*0.63*	0.1363	*0.0022*	8.9428	*0.0261*	7.8602	*0.0262*	14.9	*2.4*			
	112m	1.0807	*0.0025*	1.2009	3.73	*0.71*	0.1201	*0.0036*	9.0893	*0.0228*	8.0086	*0.0229*	12.5	*2.3*	**9.4**	77.5	
				1.1632	3.06	*0.63*	0.0824	*0.0036*	9.1104	*0.0022*	8.0296	*0.0033*	7.0	*1.4*			
	200m	1.0769	*0.0040*	1.2054	2.75	*0.71*	0.1284	*0.0057*	9.0408	*0.0147*	7.9639	*0.0152*	9.9	*2.5*	**6.3**	45	
				1.1183	2.70	*0.63*	0.0413	*0.0057*	9.0283	*0.0088*	7.9514	*0.0097*	3.1	*0.8*			**1.86**
Station C	16m	1.0811	*0.0031*	1.2200	2.70	*0.71*	0.1389	*0.0044*	9.0731	*0.0073*	7.9921	*0.0079*	10.5	*2.6*	**10.0**	25	
				1.1999	3.36	*0.63*	0.1188	*0.0044*	9.0404	*0.0037*	7.9594	*0.0048*	11.2	*2.0*			
	47m	1.0830	*0.0016*	1.1926	4.51	*0.71*	0.1097	*0.0022*	9.0056	*0.0180*	7.9226	*0.0181*	13.9	*2.1*	**10.1**	42.5	
				1.1968	2.94	*0.63*	0.1138	*0.0022*	11.1518	*0.0965*	10.0689	*0.0966*	7.4	*1.5*			
	104m	1.0800	*0.0025*	1.2014	3.47	*0.71*	0.1214	*0.0036*	9.1150	*0.0017*	8.0349	*0.0030*	11.7	*2.3*	**12.5**	77.5	
				1.2282	3.61	*0.63*	0.1482	*0.0036*	9.1354	*0.0006*	8.0553	*0.0026*	14.8	*2.5*			
	201m	1.0783	*0.0040*	1.1822	2.46	*0.71*	0.1039	*0.0057*	9.0884	*0.0024*	8.0102	*0.0047*	7.1	*2.1*	**5.8**	55	
				1.1273	3.26	*0.63*	0.0490	*0.0057*	9.0949	*0.0023*	8.0166	*0.0047*	4.5	*1.0*			**1.97**
Station D	14m	1.0837	*0.0031*	1.4568	4.26	*0.71*	0.3730	*0.0044*	9.2447	*0.0013*	8.1610	*0.0034*	43.4	*6.8*	**37.1**	25	
				1.4137	3.95	*0.63*	0.3300	*0.0044*	9.2460	*0.0053*	8.1622	*0.0062*	35.6	*5.3*			
	48m	1.0836	*0.0016*	1.3217	5.21	*0.71*	0.2381	*0.0022*	9.0999	*0.0005*	8.0162	*0.0017*	34.5	*4.4*	**27.4**	37.5	
				1.2940	4.03	*0.63*	0.2104	*0.0022*	9.2061	*0.0025*	8.1224	*0.0030*	23.3	*3.4*			
	89m	1.0809	*0.0025*	1.2684	4.73	*0.71*	0.1874	*0.0036*	9.0517	*0.0138*	7.9708	*0.0141*	24.8	*3.5*	**19.3**	77.5	
				1.2198	4.20	*0.63*	0.1388	*0.0036*	9.0837	*0.0172*	8.0028	*0.0174*	16.2	*2.3*			
	202m	1.0759	*0.0040*	1.1552	2.42	*0.63*	0.0793	*0.0057*	9.0256	*0.0005*	7.9496	*0.0041*	5.4	*1.4*	**5.1**	60	**3.76**
Station E	11m	1.0832	*0.0031*	1.2096	5.00	*0.71*	0.1264	*0.0044*	8.2668	*0.0037*	7.1836	*0.0048*	19.6	*2.7*	**17.7**	25	
				1.2052	4.57	*0.63*	0.1220	*0.0044*	8.0977	*0.0063*	7.0144	*0.0071*	17.7	*2.4*			
	46m	1.0837	*0.0016*	1.1972	5.44	*0.71*	0.1135	*0.0022*	8.7737	*0.0023*	7.6900	*0.0028*	17.9	*2.3*	**16.4**	47.5	
				1.1814	5.47	*0.63*	0.0977	*0.0022*	8.5167	*0.0019*	7.4330	*0.0025*	16.0	*1.8*			
	90m	1.0821	*0.0025*	1.2231	8.23	*0.71*	0.1410	*0.0036*	9.2279	*0.0025*	8.1458	*0.0036*	31.7	*2.8*	**30.0**	77.5	
				1.2237	7.16	*0.63*	0.1416	*0.0036*	8.9035	*0.0454*	7.8214	*0.0454*	28.9	*2.6*			
	201m	1.0783	*0.0040*	1.2140	2.99	*0.71*	0.1357	*0.0057*	8.7446	*0.0009*	7.6663	*0.0041*	11.8	*2.8*	**8.7**	50	
				1.1409	3.12	*0.63*	0.0626	*0.0057*	8.6167	*0.0126*	7.5384	*0.0132*	5.8	*1.3*			**3.98**

POC >3 µm		t = 0 Natural POC		t=24h Incubated POC			POC Enrichment		t=24h DIC		${}^{final}A_{substrate} - {}^{t=0}A_{particle}$						
		^{13}C	*Depth 3xSD*	^{13}C	Concentration		^{13}C		^{13}C		^{13}C		**Uptake C**		**Uptake rate**	**#**	**Uptake C**
		Atom%	Atom%	Atom%	[µmol/4.5L]	*E*	Atom%	*E*	Atom%	*E*	Atom%	*E*	**[nmol/l]**	*E*	**[µmol C/m^3/d]**	**meters**	**[mmol C/m²/d]**
Station A Day	12m	1.0840	*0.0059*	1.1537	8.44	*0.68*	0.0697	*0.0083*	9.1132	*0.0342*	8.0292	*0.0347*	16.3	*2.3*	**16.9**	25	
				1.1460	9.96	*0.50*	0.0620	*0.0083*	8.9680	*0.0158*	7.8839	*0.0169*	17.5	*2.5*			
	45m	1.0847	*0.0049*	1.1853	8.70	*0.68*	0.1007	*0.0069*	9.0213	*0.0051*	7.9367	*0.0070*	24.6	*2.6*	**<u>29.1</u>**	40	
				1.2205	9.35	*0.50*	0.1358	*0.0069*	9.4909	*0.0156*	8.4062	*0.0164*	33.7	*2.5*			
	86m	1.0855	*0.0038*	1.1820	5.06	*0.68*	0.0965	*0.0054*	8.8265	*0.0036*	7.7409	*0.0052*	14.1	*2.0*	**17.6**	77.5	
				1.1777	8.25	*0.50*	0.0922	*0.0054*	8.9838	*0.0016*	7.8982	*0.0041*	21.5	*1.8*			
	207m	1.0872	*0.0042*	1.1050	5.03	*0.68*	0.0178	*0.0060*	9.4700	*0.0081*	8.3828	*0.0091*	2.4	*0.8*	**3.7**	57.5	
				1.1281	4.49	*0.50*	0.0409	*0.0060*	8.8531	*0.0020*	7.7659	*0.0047*	5.3	*0.9*			**3.16**
Station B	13m	1.0883	*0.0059*	1.2504	4.36	*0.50*	0.1622	*0.0083*	8.9326	*0.0031*	7.8443	*0.0066*	20.1	*2.4*	**17.2**	25	
				1.2377	3.83	*0.50*	0.1495	*0.0083*	8.9088	*0.0039*	7.8205	*0.0070*	16.3	*2.2*			
	46m	1.0873	*0.0049*	1.2706	5.01	*0.50*	0.1833	*0.0069*	8.9367	*0.0017*	7.8494	*0.0052*	26.1	*2.7*	**<u>25.2</u>**	52.5	
				1.2858	4.59	*0.50*	0.1985	*0.0069*	8.9428	*0.0261*	7.8555	*0.0266*	25.8	*2.9*			
	112m	1.0891	*0.0038*	1.1710	4.46	*0.50*	0.0819	*0.0054*	9.0893	*0.0228*	8.0002	*0.0231*	10.2	*1.3*	**13.2**	77.5	
				1.1835	6.61	*0.50*	0.0944	*0.0054*	9.1104	*0.0022*	8.0213	*0.0044*	17.3	*1.6*			
	200m	1.0891	*0.0042*	1.1414	2.38	*0.50*	0.0523	*0.0060*	9.0408	*0.0147*	7.9517	*0.0153*	3.5	*0.8*	**9.3**	45	
				1.1631	7.69	*0.50*	0.0740	*0.0060*	9.0283	*0.0088*	7.9392	*0.0097*	16.0	*1.6*			**3.20**
Station C	16m	1.0888	*0.0059*	1.3323	4.15	*0.50*	0.2435	*0.0083*	9.0731	*0.0073*	7.9843	*0.0094*	28.2	*3.3*	**<u>30.0</u>**	25	
				1.3097	5.90	*0.50*	0.2209	*0.0083*	9.0404	*0.0037*	7.9516	*0.0069*	36.6	*3.1*			
	47m	1.0880	*0.0049*	1.2701	5.57	*0.50*	0.1822	*0.0069*	9.0056	*0.0180*	7.9176	*0.0187*	28.6	*2.6*	**24.0**	42.5	
				1.3218	4.25	*0.50*	0.2339	*0.0069*	11.1518	*0.0965*	10.0639	*0.0967*	22.0	*2.5*			
	104m	1.0863	*0.0038*	1.2089	4.57	*0.50*	0.1226	*0.0054*	9.1150	*0.0017*	8.0287	*0.0041*	15.5	*1.7*	**14.1**	77.5	
				1.2067	4.32	*0.50*	0.1204	*0.0054*	9.1354	*0.0006*	8.0491	*0.0038*	14.4	*1.7*			
	201m	1.0899	*0.0042*	1.1501	2.05	*0.50*	0.0602	*0.0060*	9.0884	*0.0024*	7.9985	*0.0048*	3.4	*0.9*	**2.6**	55	
				1.1208	2.11	*0.50*	0.0308	*0.0060*	9.0949	*0.0023*	8.0050	*0.0048*	1.8	*0.6*			**3.01**
Station D	14m	1.0890	*0.0059*	1.5900	4.71	*0.50*	0.5010	*0.0083*	9.2447	*0.0013*	8.1557	*0.0060*	64.5	*6.5*	**<u>64.5</u>**	25	
				1.5961	5.27	*0.50*	0.5070	*0.0083*	9.2460	*0.0053*	8.1569	*0.0079*	73.1	*6.6*			
	48m	1.0887	*0.0049*	1.3371	6.74	*0.50*	0.2485	*0.0069*	9.0999	*0.0005*	8.0112	*0.0049*	46.6	*3.5*	**47.7**	37.5	
				1.3779	6.80	*0.50*	0.2892	*0.0069*	9.2061	*0.0025*	8.1174	*0.0055*	54.0	*4.0*			
	89m	1.0877	*0.0038*	1.0952	1.12	*0.50*	0.0074	*0.0054*	9.0517	*0.0138*	7.9640	*0.0143*	0.2	*0.2*	**36.2**	77.5	
				1.3138	6.10	*0.50*	0.2261	*0.0054*	9.0837	*0.0172*	7.9960	*0.0176*	38.5	*3.1*			
	202m	1.0909	*0.0042*	1.1543	2.65	*0.50*	0.0635	*0.0060*	9.0256	*0.0005*	7.9347	*0.0042*	4.7	*0.9*	**4.4**	60	**6.47**
Station E	11m	1.0892	*0.0059*	1.3386	7.25	*0.50*	0.2494	*0.0083*	8.2668	*0.0037*	7.1776	*0.0069*	56.2	*4.1*	**<u>51.8</u>**	25	
				1.3414	6.58	*0.50*	0.2522	*0.0083*	8.0977	*0.0063*	7.0084	*0.0086*	52.8	*4.2*			
	46m	1.0894	*0.0049*	1.2784	5.71	*0.50*	0.1890	*0.0069*	8.7737	*0.0023*	7.6843	*0.0054*	31.3	*2.9*	**30.6**	47.5	
				1.2588	6.27	*0.50*	0.1695	*0.0069*	8.5167	*0.0019*	7.4274	*0.0053*	31.9	*2.8*			
	90m	1.0876	*0.0038*	1.1733	7.21	*0.50*	0.0857	*0.0054*	9.2279	*0.0025*	8.1403	*0.0045*	16.9	*1.6*	**22.6**	77.5	
				1.2182	7.71	*0.50*	0.1306	*0.0054*	8.9035	*0.0454*	7.8158	*0.0455*	28.7	*2.2*			
	201m	1.0909	*0.0042*	1.1471	4.35	*0.50*	0.0561	*0.0060*	8.7446	*0.0009*	7.6536	*0.0043*	7.1	*1.1*	**6.8**	50	
				1.1347	5.15	*0.50*	0.0438	*0.0060*	8.6167	*0.0126*	7.5258	*0.0133*	6.7	*1.1*			**4.84**
