# Supplementary material for: Importance of N2-Fixation on the Productivity at the North-Western Azores Current/Front System, and the Abundance of Diazotrophic Unicellular Cyanobacteria
Source: PLoS One. 2016 Mar 9;11(3):e0150827. doi: 10.1371/journal.pone.0150827 (PMC4784884; doi:10.1371/journal.pone.0150827)
Supplement: S3 Table — Corrected (i.e. given the value of natural SD/2) if <Depth 3xSD. Flagged in grey if <0.0908 (highest error from 15N2 replicates). Flagged in black if N2 fixation <propagated error E. (PDF) [file pone.0150827.s007.pdf]

**POC**	**<3µm**	**t = 0 Natural POC**		**t=24h Incubated POC**			**POC Enrichment**		**t=24h DIC**		**$^{final}A_{substrate} - ^{t=0}A_{particle}$**						
		^{13}C	*Depth 3xSD*	^{13}C	Concentration		^{13}C		^{13}C		^{13}C		**Uptake C**		**Uptake rate**	**#**	**Uptake C**
		Atom%	Atom%	Atom%	[µmol/4.5L]	*E*	Atom%	*E*	Atom%	*E*	Atom%	*E*	**[nmol/l]**	*E*	**[µmol C/m³/d]**	**meters**	**[mmol C/m²/d]**
Station A Night	14m	1.0825	*0.0031*	1.0825	3.40	*0.71*	0.1051	*0.0044*	9.3719	*0.0140*	8.2894	*0.0143*	9.6	*2.0*	**7.7**	25	
				1.1447	3.47	*0.63*	0.0623	*0.0044*	9.0193	*0.0010*	7.9368	*0.0033*	6.1	*1.2*			
	45m	1.0826	*0.0016*	1.0826	3.67	*0.71*	0.0961	*0.0022*	8.9845	*0.0022*	7.9019	*0.0027*	10.0	*1.9*	**11.3**	42.5	
				1.1885	4.19	*0.63*	0.1059	*0.0022*	8.9509	*0.0040*	7.8684	*0.0043*	12.6	*1.9*			
	90m	1.0822	*0.0025*	1.0822	4.77	*0.71*	0.1514	*0.0036*	9.0601	*0.0029*	7.9779	*0.0038*	20.2	*3.1*	**20.8**	77.5	
				1.2451	4.64	*0.63*	0.1630	*0.0036*	9.0744	*0.0283*	7.9923	*0.0284*	21.1	*2.9*			
	217m	1.0774	*0.0040*	1.0774	1.98	*0.71*	0.0551	*0.0057*	8.9998	*0.0075*	7.9224	*0.0085*	3.1	*1.2*	**7.1**	55	
				1.2131	2.84	*0.63*	0.1357	*0.0057*	8.9281	*0.0066*	7.8507	*0.0077*	10.9	*2.5*			**2.67**
	**>3µm**																
Station A Night	14m	1.0875	*0.0059*	1.2303	5.52	*0.68*	0.1427	*0.0083*	9.3719	*0.0140*	8.2843	*0.0152*	21.2	*2.8*	**18.4**	25	
				1.2055	4.92	*0.50*	0.1180	*0.0083*	9.0193	*0.0010*	7.9317	*0.0060*	16.3	*2.0*			
	45m	1.0870	*0.0049*	1.2521	6.37	*0.68*	0.1651	*0.0069*	8.9845	*0.0022*	7.8975	*0.0054*	29.7	*3.4*	**29.9**	42.5	
				1.2944	5.13	*0.50*	0.2074	*0.0069*	8.9509	*0.0040*	7.8640	*0.0063*	30.2	*3.1*			
	90m	1.0867	*0.0038*	1.2209	6.97	*0.68*	0.1342	*0.0054*	9.0601	*0.0029*	7.9733	*0.0048*	26.2	*2.8*	**22.0**	77.5	
				1.2127	4.99	*0.50*	0.1260	*0.0054*	9.0744	*0.0283*	7.9877	*0.0286*	17.6	*1.9*			
	217m	1.0890	*0.0042*	1.1654	2.41	*0.68*	0.0763	*0.0060*	8.9998	*0.0075*	7.9108	*0.0086*	5.2	*1.5*	**6.1**	55	
				1.1549	3.69	*0.50*	0.0659	*0.0060*	8.9281	*0.0066*	7.8391	*0.0078*	6.9	*1.1*			**3.78**

**PN**	**<3µm**	**t = 0 Natural PN**				**t=24h Incubated PN**					**PN Enrichment**				**t=24h Dissolved**		**$^{final}A_{substrate} - ^{t=0}A_{particle}$**		**N_2**	**N_2 fixation rate**	
		$\delta^{15}N$		*Depth 3xSD*		$\delta^{15}N$		Concentration		C:N	$\delta^{15}N$	^{15}N [A]	**Corrected**		**N_2 ^{15}N**		^{15}N		**fixation**		
		[‰]	*E*	[‰]	Atom%	[‰]	*E*	[µmol/4.5L]	*E*	ratio	[‰]	Atom%	*E*	Atom%	Atom%	*SD*	Atom%	*E*	**[nmol/L]**	**[µmol N/m³/d]**	*E*
Station A Night	14m	0.26	*1.05*	*3.40*	*0.0012*	5.65	*2.48*	0.36	*0.12*	9.40	5.39	0.0020	*0.0006*	0.0020	0.7410	*0.0879*	0.3746	*0.0879*	0.424	0.415	0.209
						8.82	*0.79*	0.28	*0.05*	12.60	8.56	0.0031	*0.0006*	0.0031	0.9440	*0.0908*	0.5776	***0.0908***	0.333	0.326	0.102
	45m	-0.56	*1.01*	*13.85*	*0.0051*	5.87	*2.42*	0.37	*0.12*	9.87	6.43	0.0023	*0.0024*	0.0008	0.7302	*0.0338*	0.3641	*0.0338*	0.192	0.192	0.547
						10.00	*0.59*	0.41	*0.05*	10.13	10.56	0.0039	*0.0024*	0.0008	0.8441	*0.0312*	0.4780	*0.0312*	0.163	0.163	0.461
	90m	2.03	*0.70*	*2.69*	*0.0010*	5.86	*1.44*	0.64	*0.12*	7.43	3.83	0.0014	*0.0005*	0.0014	0.5990	*0.0020*	0.2319	*0.0021*	0.863	0.869	0.331
						7.49	*0.41*	0.63	*0.05*	7.34	5.46	0.0020	*0.0005*	0.0020	0.4601	*0.0033*	0.0931	*0.0033*	3.018	3.039	0.760
	217m	9.14	*2.46*	*13.14*	*0.0048*	6.21	*5.78*	0.14	*0.12*	14.05	-2.93	-0.0011	*0.0023*	0.0008	0.7015	*0.0065*	0.3318	*0.0067*	0.076	0.077	0.226
						12.90	*0.75*	0.30	*0.05*	9.35	3.76	0.0014	*0.0023*	0.0008	0.4311	*0.0004*	0.0615	*0.0016*	0.880	0.891	2.524
	**>3µm**																				
Station A Night	14m	2.15	*0.47*	*2.44*	*0.0009*	4.05	*0.61*	0.68	*0.12*	8.08	1.90	0.0007	0.0004	0.0001	0.7410	*0.0879*	0.3739	***0.0879***	0.060	0.059	*0.040*
						3.34	*0.38*	0.57	*0.10*	8.64	1.18	0.0004	0.0004	0.0001	0.9440	*0.0908*	0.5769	*0.0908*	0.033	0.032	*0.032*
	45m	1.12	*0.46*	*4.62*	*0.0017*	2.69	*0.49*	0.82	*0.12*	7.75	1.57	0.0006	0.0008	0.0003	0.7302	*0.0338*	0.3634	*0.0338*	0.142	0.142	*0.198*
						2.23	*0.31*	0.69	*0.10*	7.46	1.11	0.0004	0.0008	0.0003	0.8441	*0.0312*	0.4774	*0.0312*	0.090	0.090	*0.178*
	90m	2.92	*0.40*	*1.74*	*0.0006*	5.62	*0.37*	1.10	*0.12*	6.31	2.70	0.0010	0.0003	0.0010	0.5990	*0.0020*	0.2316	*0.0020*	1.048	1.055	*0.339*
						3.52	*0.29*	0.76	*0.10*	6.55	0.60	0.0002	0.0003	0.0001	0.4601	*0.0033*	0.0927	*0.0033*	0.194	0.195	*0.269*
	217m	0.40	*1.66*	*6.92*	*0.0025*	24.74	*1.56*	0.43	*0.12*	5.55	24.34	0.0089	0.0012	0.0089	0.7015	*0.0065*	0.3350	*0.0066*	2.565	2.597	*0.777*
						4.95	*0.42*	0.54	*0.10*	6.88	4.55	0.0017	0.0012	0.0004	0.4311	*0.0004*	0.0647	*0.0009*	0.780	0.790	*0.587*
