# Supplementary material for: Importance of N2-Fixation on the Productivity at the North-Western Azores Current/Front System, and the Abundance of Diazotrophic Unicellular Cyanobacteria
Source: PLoS One. 2016 Mar 9;11(3):e0150827. doi: 10.1371/journal.pone.0150827 (PMC4784884; doi:10.1371/journal.pone.0150827)
Supplement: S4 Table — Spearman correlations’ significance at p<0.001, p<0.01 and p<0.05 are shown with ***, ** and *, respectively. O2: oxygen saturation %; Temp: potential temperature; Sal: salinity; PAR: % surface photoactive radiation; Chl: in situ Chlorophyll fluorescence. POM, POC, PN: Particulate Organic Matter, Carbon, Nitrogen concentrations; C-Fix, N2-Fix: C- and N2-fixation and Pico-UCYN abundance are examined for the small (<3 μm, s) and larger (>3 μm, L) size fractions. (PDF) [file pone.0150827.s008.pdf]

	Environmental Variables		Correlation	POM concentration		Correlation	Activity/UCYN abundance		Correlation
**Surface**	O_2	Silicate	(-)0.81**	POC*s*	PN*s*	0.91***	C-Fix *s*	C-Fix *L*	0.95***
	Temp	Silicate	(-)0.75*		PN_L	0.74*			
				POC_L	PN_L	0.90***	Pico-UCYN *s*	Sal	1.00***
**Above DCM**	Sal	O_2	(-)1.00***	POC*s*	PN*s*	0.90***	C-Fix *s*	Sal	(-)0.89*
	Sal	Silicate	(-)0.78*	POC_L	PN_L	0.89***		O_2	0.89*
	O_2	Silicate	0.78*	PN*s*	PN_L	0.76*			
	PAR	Temp	(-)0.75*						
**DCM**	PAR	Silicate	0.89***	POC*s*	Chl	0.82**	C-Fix *s*	PN*s*	0.94**
	Sal	Chl	(-)0.89**	POC_L	POC*s*	0.80*		POC*s*	0.92**
	Temp	Sal	0.83**	PN*s*	POC*s*	0.90***			
	O_2	Nitrate	(-)0.78*		Chl	0.80*			
	Temp	Phosphate	(-)0.77*						
**Mesopelagic**	Temp	Sal	1.00***	PN_L	POC_L	0.78*	N_2 fix *s* not detected		
	Nitrate	Silicate	1.00***						
	Temp	Nitrate	(-)0.93***						
	Temp	Silicate	(-)0.93***						
	Sal	Nitrate	(-)0.93***						
	Sal	Silicate	(-)0.93***						
	Ammonium	Nitrate	0.89***						
	Ammonium	Silicate	0.89***						
