# Supplementary material for: Importance of N2-Fixation on the Productivity at the North-Western Azores Current/Front System, and the Abundance of Diazotrophic Unicellular Cyanobacteria
Source: PLoS One. 2016 Mar 9;11(3):e0150827. doi: 10.1371/journal.pone.0150827 (PMC4784884; doi:10.1371/journal.pone.0150827)
Supplement: S5 Table — (PDF) [file pone.0150827.s009.pdf]

**Location**	**Depth**	**Period**	**N_2-fixation volumetric rates (µmol N m^{-3} d^{-1})**	**Reference**
31.5°N, 33.0°W-36.2°N,33.9°W	11-217m	Early August 2011	0.2-7.9	This study
35.5°N,29.5°W	0-100m	Late October 2001	0.07-0.09	Moore *et al.*, 2009
29.0°N,29.0°W	0-100m	February 2006	0.01-0.03	Fernandes *et al.*, 2010
29.0°N,29.0°W	0-100m	May 2008	0.01-0.03	Rijkenberg *et al.*, 2011
35.8°N,75.4°W-37.9°N,38.6°W	2-29m	August 2009	0.5-9.0	Pangaea database, Luo *et al.*, 2012
34-36°N,28.5°W	0-60m	September 2006	0.8-1.7	Krupke *et al.*, 2014
