# Supplementary material for: Importance of N2-Fixation on the Productivity at the North-Western Azores Current/Front System, and the Abundance of Diazotrophic Unicellular Cyanobacteria
Source: PLoS One. 2016 Mar 9;11(3):e0150827. doi: 10.1371/journal.pone.0150827 (PMC4784884; doi:10.1371/journal.pone.0150827)
Supplement: S1 Text — (PDF) [file pone.0150827.s010.pdf]

Supporting Online Material (SOM) for:

Impact of contrasting conditions at the North-Western Azores Current on carbon- and N_2-fixation rates and the abundance of diazotrophic unicellular cyanobacteria

Virginie Riou [1,2,+], Debany Fonseca Batista [2,3], Arnout Roukaerts [3], Isabelle C. Biegala [2] ,

Shree Ram Prakya [1], Clara Magalhães Loureiro [4,7], Mariana Santos [5,6], Angel E.M. Muniz-Piniella [3],

Mara Schmiing [1,6,7], Marc Elskens [3], Natacha Brion [3], M. Ana Martins [4,7], Frank Dehairs [3]

[1] IMAR—Institute of Marine Research, Centre of IMAR at the University of the Azores, Horta, Portugal
[2] Aix-Marseille Université, Mediterranean Institute of Oceanography (MIO), 13288 Marseille; Université du Sud Toulon-Var, 83957 La Garde, UM 110 CNRS/INSU, IRD, France
[3] Analytical, Environmental and Geo-Chemistry & Earth System Sciences, Vrije Universiteit Brussel, Brussels, Belgium
[4] CIBIO, Research Center in Biodiversity and Genetic Resources, InBIO Associated Laboratory, Department of Oceanography and Fisheries, Horta, Portugal
[5] IPMA, I.P.—Portuguese Institute of Ocean and Atmosphere, Lisbon, Portugal
[6] MARE—Marine and Environmental Sciences Centre, Lisbon, Portugal
[7] DOP/UAz – Department of Oceanography and Fisheries, University of the Azores, Azores, Portugal

Materials and Methods:
During the DIAPICNA cruise (25 July-3 August 2011) aboard R.V. "NRP Dom Carlos I", five stations were sampled from 31.5°N-33.0°W to 36.2°N-33.9°W (Station A: 31°29'53''N, 33°00'01''W, 4360 m bottom depth (b.d.); Station B: 32°11'58''N, 33°00'01''W, 3740 m b.d.; Station C: 33°42'01''N, 32°59'57''W, 3324 m b.d.; Station D: 35°00'00''N, 33°00'02''W, 3091 m b.d.; Station E: 36°13'58''N, 33°54'02''W, 2334 m b.d.). At each station, the water column physical properties were recorded down to 500-2000m depth using *in situ* sensors. The upper 500m of the water column was sampled at 12 discrete depths for the measurement of nutrient concentrations. Particulate nitrogen (PN) and organic carbon (POC) concentrations, natural NO_3^- isotopic signatures, carbon- and N_2 fixation rates and the abundance of Unicellular CYanobacterial N_2-fixers (UCYN) were assessed on water samples sequentially collected during four additional casts at four chosen depths within the euphotic zone. All longitudinal cross-sections were obtained using the Ocean Data View software (Schlitzer, 2008).

N_2- and C-fixation incubations: N_2 fixation rates were measured using the dissolved $^{15}N_2$ tracer method (Mohr *et al.*, 2010; Wilson *et al.*, 2012). Spiked solutions of $^{15}N_2$ were prepared prior to the cruise, using degassed (by vacuum pumping under magnetic stirring) 0.2 µm-filtered low nutrient seawater (Osil, UK), injected with 2.5 mL of $^{15}N_2$-gas (98+ Atom% ^{15}N, Eurisotop, France) and shaken for 24 h. These spiked solutions were conditioned in 285 mL borosicilate vials crimp sealed with Teflon lined silicone septa (Wheaton, US).

Prior to the cruise, tests were conducted in the lab using five replicate spiked solutions in separate 285 mL borosilicate vials sampled respectively after 0, 2, 4, 7 and 9 days incubation at room temperature. They showed that after an initial decrease from 70% to 50% ^{15}N atom% between days 0 and 4, the 50% enrichment remained stable for up to 9 days. Final enrichment in incubation bottles were also conducted in triplicates: three acid-washed 4.5-L Nalgene polycarbonate bottles were filled with 4.3L of non-degassed 0.2 µm-filtered low nutrient seawater (OSIL incubation seawater) to which 285 mL $^{15}N_2$ spiked solutions (previously homogenised for 24h) were added. Nalgene bottles were filled to the very rim with the OSIL incubation seawater, capped with septum equipped screw caps (PTFE; Nalgene) and incubated for 24 h at room temperature (23°C) in the lab, before they were sampled for dissolved N_2 ^{15}N atom%. Triplicate 12 mL aliquots were transferred from each incubation

bottle through the PTFE septa, applying a slight He overpressure and using 2-way needles, into helium pre-flushed exetainers (Labco, UK) poisoned with 100 µL saturated $HgCl_2$ solution. Dissolved N_2 isotopic composition was measured from equilibrated headspaces prepared by the removal of 3 mL seawater from the 12 mL exetainer vials, using He pressure. Analyses were performed using a Flash EA 1112 Elemental Analyzer coupled to a DELTA V Isotope Ratio Mass Spectrometer via a Conflo III interface (EA-IRMS, Thermo Instruments) equipped with a custom made manual gas injection port. The injections of 300 µL headspace were done upstream of the EA reduction column, under helium flow. These analyses revealed final dissolved N_2 ^{15}N atom% of 5.4 ± 0.4 % in the incubated seawater.

During the cruise, seawater samples were taken at each station at the surface (11-16m), above the Deep Chlorophyll Maximum (DCM; 45-48m), at the DCM (86-112m) and in the upper mesopelagic zone (200-217m), from which 4.5 L were immediately filtered to determine natural particulate organic Carbon and Nitrogen (POC, PN) concentrations and their ^{13}C and ^{15}N isotopic compositions. Further duplicate samples from each depth (approx. 4.3 L) were transferred to acid-washed 4.5-L Nalgene polycarbonate bottles to which 285 mL $^{15}N_2$ spike solution were added, together with 0.73 mmol of dissolved $NaH^{13}CO_3$ each (99 Atom% ^{13}C, Eurisotop, France), to reach theoretical enrichments of 5 Atom% ^{15}N and 10 Atom% ^{13}C. Nalgene bottles were then filled to the very rim with ambient seawater and capped with a screw cap fitted with a septum (PTFE; Nalgene). These spiked samples were incubated for 24 h in on-deck incubators wrapped in blue filters (Rosco, UK) and bathed in continuously renewed surface seawater to ascertain a constant temperature (around 24°C). The light filters were selected to mimic *in situ* daylight attenuation in Jerlov type OIA waters (Piazena *et al.*, 2002): no filter for surface samples (100% surface light), "No color Blue" filter for above the DCM samples (#144 ≈30% light transmission), "Dark Blue" filter for DCM samples (around 100 m, #119≈3% light transmission) and an overlap of "Alice Blue" and "Tokyo Blue" filters for upper mesopelagic samples (#197+#071≈0.1% light transmission).

At the end of the incubation period, six replicate 12 mL aliquots were transferred through the PTFE septa, applying a slight He overpressure and using 2-way needles, into helium pre-flushed exetainers (Labco, UK) poisoned with 100 µL saturated $HgCl_2$ solution for the assessment of the dissolved N_2 ^{15}N atom% and Dissolved Inorganic Carbon (DIC) ^{13}C atom%. Triplicate dissolved N_2 and DIC isotopic compositions were measured from equilibrated headspaces prepared by the removal of 3 mL seawater from the 12 mL exetainer vials, using He pressure. For DIC we subsequently added 200µl of 99% phosphoric acid. Analyses were performed using a Flash EA 1112 Elemental Analyzer coupled to a DELTA V Isotope Ratio Mass Spectrometer via a Conflo III interface (EA-IRMS, Thermo Instruments) equipped with a custom made manual gas injection port. The injections of 300 µL headspace were done upstream of the EA reduction column, under helium flow.

Natural and enriched particles were size-fractionated by serial filtration onto 25 mm diameter membranes of 3.0 µm and 0.3 µm porosities, made of silver (Sterlitech) and pre-combusted glass fiber (GF75, Advantec MFS Inc.), respectively. The filters were dried overnight at 60°C and stored over desiccant until analysis. After carbonate acidification overnight under saturated HCl vapours and drying at 60°C, POC and PN concentrations and $\delta^{13}C$ and $\delta^{15}N$ signatures were measured using the EA-IRMS. Standards and blanks were run under the same analytical conditions as the samples. POC and PN areas were corrected for blank areas and converted to moles using acetanilide standard calibration (Merck, 71.09%C, 10.36%N). IRMS $\delta^{13}C$ and $\delta^{15}N$ values were corrected for the influence of blanks and for the offset of the IAEA standards from their certified values (IAEA-CH6, $\delta^{13}C$=+10.449‰ vs. Vienna Pee Dee Belemnite; IAEA-N1, $\delta^{15}N$=+0.43‰ vs. atmospheric N_2). The uncertainties associated with the calibrations are reported in Tables S1, S2 and S3 under "t=0 Natural PN, $\delta^{15}N$, *E*", "t=24h Natural PN, $\delta^{15}N$, *E*" and "t=24h Natural PX, Concentration, *E*".

$\delta^{H}X$ values (i.e. $\delta^{15}N$ or $\delta^{13}C$) were converted to ^{H}X atom% using the following formulas:

$R = (^{H}X/^{L}X)_{sample} = [(\delta^{H}X/1000)+1] \times (^{H}X/^{L}X)_{reference}$ and $^{H}X \text{ atom\%} = R/[1+R]$

Where H is the heavy and L is the light isotope and references are atmospheric N_2 and Vienna Pee Dee Belemnite for N and C, respectively.

N_2- and C-fixation rates and error calculations: The $^{final}A_{substrate}$ term for DIC was corrected by the equation of Miyajima *et al.* (1995), and that for dissolved $^{15}N_2$ was calculated using the combination of IRMS masses M_{28} ($^{14}N_2$), M_{29} ($^{14}N^{15}N$) and M_{30} ($^{15}N_2$) areas (after correction of the machine bias for M_{30}):

$^{final}A_{N2} = [M_{30} + (M_{29}/2)]/[M_{28} + M_{29} + M_{30}]$

N_2 and C-uptake rates were not considered significant (grey police in Tables S1, S2 and S3) when:

(i) particle enrichment ("PX Enrichment") was lower than three times the standard deviation of the natural PX isotopic signature calculated for each depth ("t=0 Natural PX *Depth 3xSD*"), and/or the particle enrichment after incubation in atom% was lower than the error calculated on the enrichment ("PX enrichment *E*");

(ii) Substrate enrichment in atom% ("Substrate enrichment") was lower than the highest error on $^{final}A_{substrate}$ triplicate measurements (i.e., $^{15}N_2$<0.0908);

(iii) The error on the uptake rate calculation ("Uptake Rate *E*"), calculated from the propagation of the errors or uncertainties associated with each term used for the calculation, was higher than the uptake rate value itself.

Unicellular cyanobacterial diazotroph (UCYN) cellular abundance: Cells were collected on the 0.2, 3.0 and 10 µm porosity PCTE membranes, applying light vacuum as for the POC/PN filters with a water jet pump (Medline, Model VE-11) reaching an average filtration speed of 100 mL min^{-1}. Collected cells were then fixed with 10 mL of 1% paraformaldehyde (pH 7.2 buffered with PBS and clarified by filtration, Sigma-Aldrich, France) for 15 min at room temperature (RT), desiccated in 100% ethanol molecular grade (Sigma-Aldrich, France) for 10 min at RT, and immediately preserved in liquid N_2 until the end of the cruise. During transfer and at the lab, filters were stored between -20°C and -80°C until analysis.

Nitrate dual isotopic composition ($\delta^{15}N$ and $\delta^{18}O$) measurements: Isotope signatures for nitrate were measured using the denitrifier method (Sigman *et al.*, 2001; Casciotti *et al.*, 2002). Nitrate was converted to N_2O by adding sample to a solution of denitrifying bacteria (*Pseudomonas aureofaciens*) that lack the active N_2O reductase. The produced N_2O was then cryogenically isolated and purified from the sample headspace for subsequent isotopic analysis for $\delta^{15}N$ and $\delta^{18}O$ using IRMS (DELTA, Thermo Instruments). Nitrate concentrations down to 1 µmol L^{-1} could be analysed.

Results:

C-fixation integration from <3µm + >3µm size fractions

StA: day 2.42+3.16=5.58 mmolC m^{-2} d^{-1}=66.96 mgC m^{-2} d^{-1}; 43% <3µm
night 2.67+3.78=6.45 mmolC m^{-2} d^{-1}=77.4 mgC m^{-2} d^{-1}; 41% <3µm
StB: day 1.86+3.20=5.06 mmolC m^{-2} d^{-1}=60.72 mgC m^{-2} d^{-1}; 37% <3µm
StC: day 1.97+3.01=4.98 mmolC m^{-2} d^{-1}=59.76 mgC m^{-2} d^{-1}; 40% <3µm
StD: day 3.76+6.47=10.23 mmolC m^{-2} d^{-1}=122.16 mgC m^{-2} d^{-1}; 37% <3µm
StE: day 3.98+4.84=8.82 mmolC m^{-2} d^{-1}=105.84 mgC m^{-2} d^{-1}; 45% <3µm

N_2-fixation measurements and corrections

The decrease in $^{15}N_2$ final incubation enrichment level from 5.0 ^{15}N Atom% for the test incubations prior to the cruise, down to 0.4-1.0 ^{15}N Atom% for the samples incubated during the cruise (S5 & S7 Tables) indicates that the Teflon-lined silicon septa used to seal the serum bottles with $^{15}N_2$ spike did not remain gas tight over prolonged periods. This caused loss of $^{15}N_2$ during transportation and storage before the cruise. This problem was solved *a posteriori*, using bromobuthyl septa that avoided the loss of $^{15}N_2$ enrichment for up to 2 months at 5-30°C. We did not take into consideration the incubations with substrate enrichments lower than 0.0908% above $^{t=0}A_{particle}$, which was the largest measurement error on replicates for dissolved N_2 ^{15}N Atom%. Some samples were 'corrected' for insufficient particle enrichment (i.e. lower than 3 times the natural $\delta^{15}N_{PN}$ standard deviation)

using the value of half the standard deviation of natural isotopic signatures from all stations at the same depth (Tables S1 and S3). Since resulting corrected N_2 fixation rates presenting uncertainties (*E*) inferior to the value itself could reach values of 0.5±0.4 µmol N m^{-3} d^{-1}, rates below this value should be considered with care.

References:

Casciotti, K.L., Sigman, D.M., Hastings, M.G., Böhlke, J.K., Hilkert, A., 2002. Measurement of the oxygen isotopic composition of nitrate in seawater and freshwater using the denitrifier method. Anal. Chem. 74(19), 4905-4912.

Krupke, A., Lavik, G., Halm, H., Fuchs, B.M., Amann, R.I., Kuypers, M.M.M., 2014. Distribution of a consortium between unicellular algae and the N_2 fixing cyanobacterium UCYN-A in the North Atlantic Ocean. Environ. Microbiol. 16(10), 3153-3167. doi:10.1111/1462-2920.12431

Luo, Y.W., Doney, S.C., Anderson, L.A., Benavides, M., Bode, A., Bonnet, S., *et al.*, 2012. Database of diazotrophs in global ocean: abundances, biomass and nitrogen fixation rates. Earth Syst. Sci. Data Discuss. 5, 47-106. Pangaea database

Miyajima, T., Yamada, Y., Hanba, Y., Yoshii, K., Koitabashi, T., Wada, E., 1995. Determining the stable-isotope ratio of total dissolved inorganic carbon in lake water by GC/C/IRMS. Limnol. Oceanogr. 40(5), 994-1000.

Mohr, W., Grosskopf, T., Wallace, D.W.R., LaRoche, J., 2010. Methodological underestimation of oceanic nitrogen fixation rates. PLoS ONE 5, e12583.

Moore, C.M., Mills, M.M., Achterberg, E.P., Geider, R.J., LaRoche, J., Lucas, M.I., *et al.*, 2009. Large-scale distribution of Atlantic nitrogen fixation controlled by iron availability. Nature Geosci. 2, 867-871.

Piazena, H., Perez-Rodrigues, E., Häder, D.P., Lopez-Figueroa F., 2002. Penetration of solar radiation into the water column of the central subtropical Atlantic Ocean—optical properties and possible biological consequences. Deep-Sea Res. II 49, 3513-3528.

Rijkenberg, M.J.A., Langlois, R.J., Mills, M.M., Patey, M.D., Hill, P.G., Nielsdóttir, M.C., *et al.*, 2011. Environmental Forcing of Nitrogen Fixation in the Eastern Tropical and Sub-Tropical North Atlantic Ocean. PLoS ONE 6(12), e28989. doi:10.1371/journal.pone.0028989

Schlitzer (2004) Ocean Data View, http://odv.awi-bremerhaven.de

Sigman, D.M., Casciotti, K.L., Andreani, M., Barford, C., Galanter, M., Böhlke, J.K., 2001. A bacterial method for the nitrogen isotopic analysis of nitrate in seawater and freshwater. Anal. Chem. 73(17), 4145–4153, doi:10.1021/ac010088e.

Wilson, S.T., Böttjer, D., Church, M.J., Karl, D.M., 2012. Comparative assessment of nitrogen fixation methodologies conducted in the oligotrophic North Pacific Ocean. Appl. Environ. Microbiol. 78, 6516-6523.
